# Organization of feed-forward loop motifs reveals architectural principles in natural and engineered networks

**DOI:** 10.1126/sciadv.aap9751

**Published:** 2018-03-28

**Authors:** Thomas E. Gorochowski, Claire S. Grierson, Mario di Bernardo

**Affiliations:** 1 BrisSynBio, Life Sciences Building, Bristol BS8 1TQ, UK.; 2 School of Biological Sciences, University of Bristol, Life Sciences Building, Tyndall Avenue, Bristol BS8 1TQ, UK.; 3 Department of Engineering Mathematics, University of Bristol, Bristol BS8 1TH, UK.; 4 Department of Electrical Engineering and Information Technology, University of Naples Federico II, Via Claudio 21, Napoli, Italy.

## Abstract

Network motifs are significantly overrepresented subgraphs that have been proposed as building blocks for natural and engineered networks. Detailed functional analysis has been performed for many types of motif in isolation, but less is known about how motifs work together to perform complex tasks. To address this issue, we measure the aggregation of network motifs via methods that extract precisely how these structures are connected. Applying this approach to a broad spectrum of networked systems and focusing on the widespread feed-forward loop motif, we uncover striking differences in motif organization. The types of connection are often highly constrained, differ between domains, and clearly capture architectural principles. We show how this information can be used to effectively predict functionally important nodes in the metabolic network of *Escherichia coli*. Our findings have implications for understanding how networked systems are constructed from motif parts and elucidate constraints that guide their evolution.

## Teaser

We develop methods to decipher the rules controlling how small structures cluster and connect in complex networks.

## INTRODUCTION

Networks are commonly used to represent the complex interactions between components found in natural and engineered systems. Making sense of these structures has so far relied on the analysis of global topological features, such as degree distributions or clustering coefficients (*1*), and the classification of significant localized structures called network motifs (*2*, *3*).

Major progress has been made in the literature toward understanding how some motifs contribute to network structure and function. This has involved proving that motifs exist, that they are not there by accident, and that they make significant functional contributions to networks (*2*–*7*). Important families of motifs that are shared by diverse networks carrying out similar functions have been discovered (*2*, *5*), and attempts have been made to relate motif structure with motif function. This earlier work has shown that motifs play an important role in gene regulation (*3*, *8*), accelerated response times (*9*), dynamic stability (*10*), and responses to noise (*11*).

Even with these detailed studies, the functional importance of motifs is often uncertain and contested (*12*, *13*). In particular, it is not clear to what extent the functions of the motifs depend on the context in which they are found (that is, their specific dynamical parameters or their position and connections within the network) (*13*). For example, Burda *et al.* (*14*) evolved gene regulatory networks in silico for user-defined functions. They found that simple network functions resulted in the emergence of motifs where each had an isolated function. However, as more complex phenotypes were chosen, the individual role of the emergent motifs became less clear. Instead, motifs acted more like parts in a larger machine, and the function of each motif could only be understood in context.

Some efforts have been made to study larger motif-based structures in complex networks. Kashtan *et al.* (*15*) developed the concept of network motif generalizations. These assume that a motif can act as a template from which larger network structures can be built, specifically through duplication of nodes and associated edges that share a similar role [for example, inputs or outputs; see Kashtan *et al*. (*15*) for a formal definition]. An example of a motif generalization is the bifan where two input nodes are connected to a set of output nodes whose number may potentially vary. In this case, a single bifan (two inputs and two outputs) would form the template motif, and variants with larger numbers of output nodes would be classified as generalizations. Generalizations of motifs often maintain the dynamical function of the template motif, and specific examples of multi-input and multi-output feed-forward loops (FFLs) were shown to be capable of testing for signal persistence and the temporal ordering of events (*15*). Although generalized motifs offer a way of classifying families of related motif, this approach neglects the many ways that a given motif can connect within a substructure that does not involve duplication or connections between motifs of completely different types [for example, FFLs connected to feedback loops (FBLs)].

Taking an alternate approach, Benson *et al.* (*16*) examined the higher-order organization of networks by finding highly interconnected communities of motifs. By combining motif analysis and network partitioning and by exploiting a number of mathematical results, these authors were able to develop fast algorithms to identify these communities in large complex networks. Although this approach allowed for clusters of motifs to be efficiently found and extracted, it treated an entire cluster as a single entity and, thus, provided no insight into their internal connection structure.

Here, we set out to discover how the diversity of connections between different motifs contributes to the formation of large statistically overrepresented structures, which form an additional type of building block in complex networks. Because previous studies have neglected these aspects, our understanding of how motifs throughout a network coordinate and tune their collective function is limited. Understanding is further hindered by current analysis methods that are fundamentally unable to capture how network motifs are connected to produce functionally important topological features at these intermediate scales. Although motif aggregation has been observed previously in several biological networks [for example, gene transcription (*17*) and protein interactions (*18*)] and shown to be interwoven with global statistical properties (*19*), there is currently no standard way to quantify the spectrum of possible connection types and extract the rules that might underlie the aggregation process.

Here, we present new tools to decipher the structure of a complex network and reveal the precise organization of connections between network motifs to detect, categorize, and quantify motif clusters. Furthermore, we study how information flows through the nodes in these structures. Focusing on the widespread and highly studied FFL motif, we investigate how structures of FFLs are organized in a range of natural and engineered networks. Our results reveal highly distinctive types of FFL cluster for different types of network. Random networks have very different distributions compared to the natural and engineered networks that we tested. Although many types of clustering are possible, often just one or two types dominate, forming more than 80% of the FFL clusters. A similar observation is made for the FBL motif. The types of motif clustering that dominate depend on the type of network. We illustrate that characterizing network structures at the scale of motif clustering produces highly distinctive and surprisingly simple profiles from which clear conclusions about network structure, function, and evolution can be drawn.

## RESULTS

A broad range of biological, engineered, and social networks were selected for analysis. These covered the transcriptional regulation of *Escherichia coli* (*4*) and *Saccharomyces cerevisiae* (*2*), Gnutella peer-to-peer file sharing (*20*), Wikipedia voting (*21*), air traffic control, European Union (EU) emails (*22*), Little Rock Lake food web (*23*), metabolism of *E. coli* and *Archaeoglobus fulgidus* (*24*), and the neural network of *Caenorhabditis elegans* (*25*) (see text S1 for further details). Although these networks displayed a broad range of global statistics (table S1), in every case, the FFL motif was found to be significantly overexpressed (*P* < 0.0001; table S2). This has been recognized previously for many of the networks (*2*, *4*, *5*, *7*, *24*) and suggests that FFLs may play a key functional role across all these systems, acting as a generic building block for many different types of complex system.

### FFLs cluster in real-world networks

**Fig. 1:**
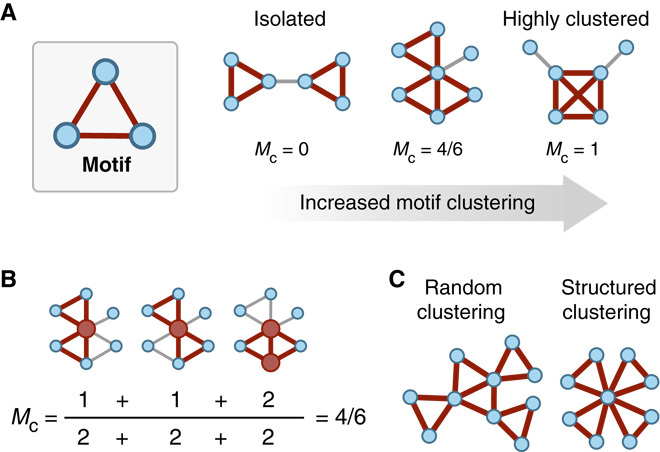
Key features of motif organization in complex networks. (**A**) The motif clustering coefficient ***M*_c_** represents the amount of overlap in terms of shared nodes between all pairs of a particular set of motifs. This example shows increasing motif clustering for a set containing a single triangular motif (highlighted in red). (**B**) An example of how the motif clustering coefficient is calculated for the intermediate network from (A). The two motifs and shared nodes have been highlighted in red, and for each pair of motifs, the maximum possible shared nodes are two (found in the denominator). (**C**) Motif clustering can occur in many different ways, be it random or structured. Further information regarding specific types of connection between motifs is required if important underlying features are to be understood (Fig. 2).

Having found FFLs to be a significant feature of the real-world networks, we next investigated their general organization by studying their propensity to aggregate and become clustered (Fig. 1A). To capture this property, we defined a measure of motif clustering (*M*_c_) calculated as the proportion of shared nodes between all pairs of FFLs, normalized by the maximum number of possible shared nodes between all pairs (Fig. 1B and Materials and Methods). A motif clustering value of 0 would correspond to each FFL in our networks being fully isolated (sharing no nodes with any others), whereas a value of 1 would represent every FFL sharing the same two common nodes, with only a single node differentiating each motif (a single fully clustered region). This measure was further generalized to allow for sets containing different types of motif (Materials and Methods; see text S2 for an example).

Analysis of the networks found FFL clustering to be a significant feature in all cases, with a positive correlation between overall motif expression and clustering (table S2). Increases in motif clustering are inevitable as the density of FFLs increases. However, it is important to note that the significance we report here relates to a null random model that maintains the same number of FFLs (Materials and Methods). The motif clustering that we see is significantly higher than we would expect given the number of FFLs present.

### Motif clustering types and their links to network function

Motif clustering alone reveals little about the specific ways in which FFLs become clustered and whether there are underlying organizational principles to how they interact (Fig. 1C). The dangers of considering only the global statistical features of a network were highlighted by Li *et al.* (*26*). They showed that, for a general statistical feature such as the degree distribution, a near-identical power law distribution could be generated by completely different types of underlying network structure. Furthermore, specific differences in the way particular nodes were connected in these networks yielded large differences in their performance and reliability to attacks, calling into question findings that often state that power law and scale-free–like networks are inherently susceptible to targeted attacks at hubs. This work clearly illustrated that the differences in local connections really do matter from both a structural and a functional perspective.

**Fig. 2:**
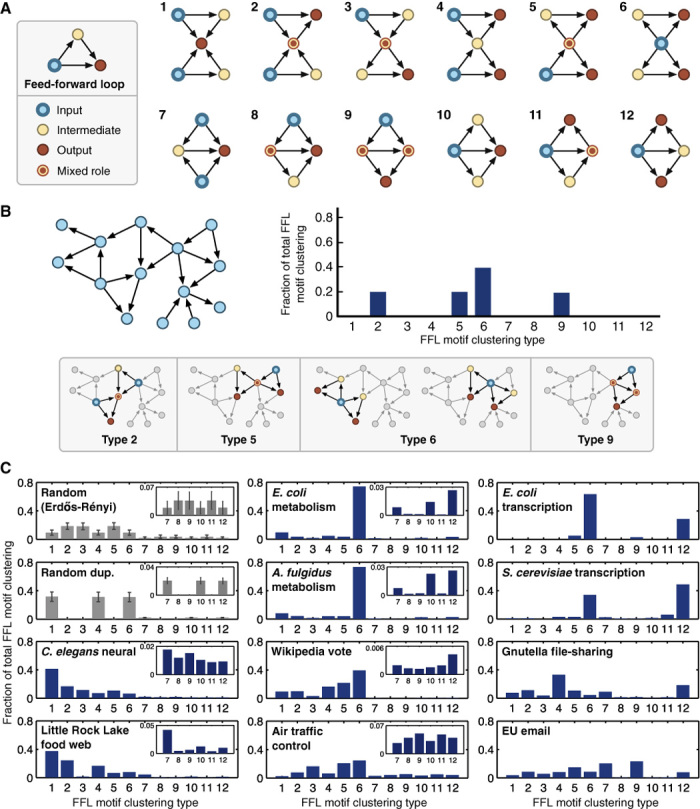
Classifying FFL motif clustering types and their distributions. (**A**) The 12 unique motif clustering types for two FFLs. To highlight the flow of information, each node has been colored in relation to its role: input, intermediate, output, or a mixture of these roles. (**B**) Example network (left) with the associated motif clustering type distribution (right), and the motif pairs and their classifications (below). (**C**) Motif clustering type distributions for the natural and engineered networks. Clear signatures are shown in the types of motif clustering found. Insets have been included for distributions where motif clustering types 7 to 12 are observed at low overall fractions. The random Erdős-Rényi distribution is generated from a network of 1000 nodes and an edge probability of 0.005 with **±**1 SD from a sample of 1000 networks. The random duplication distribution is generated from a network of 30 nodes with **±**1 SD from a sample of 1000 networks.

To ensure that we considered such localized features, we next analyzed the specific ways in which motifs were connected by categorizing the different possible pairwise combinations of (coherent) FFLs found in our networks (Fig. 2). These fall into 12 types, and we quantified the populations of each FFL combination as a fraction of the total.

Looking at the FFL clustering type distributions of the natural and engineered networks (Fig. 2C), it is apparent that it is not just clustering that matters; different types of FFL cluster are prominent in different networks, and there are similarities between networks with similar functions. This suggests that some types of FFL cluster are better suited to specific tasks than others. We investigated this further by focusing on specific types of clustering that are especially overrepresented. We hypothesized that these might be cases where one or more specific types of FFL cluster have been subjected to strong positive selection.

#### Motif clustering types in biological networks

The strongest bias in our data was seen in the two metabolic networks (figs. S1 and S2) where, in both cases, more than 70% of the FFL clustering is of type 6 (Fig. 2C). Type 6 FFL clusters have a single node that is an input to all the other nodes in the cluster (Fig. 2A). In metabolic networks, these nodes represent enzymes that produce metabolites that are consumed by multiple enzymes. The prevalence of type 6 FFL clusters reflects the broad use of the same compound by multiple biosynthetic pathways. It should be noted that the networks that we study here include cofactors such as ATP (adenosine 5′-triphosphate) and ADP (adenosine 5′-diphosphate), which are known to bind metabolism together. Their pervasive use leads to the small-world structure of the overall network (see fig. S3 for an example of this closely knit architecture with some of the most highly connected node identities included). Furthermore, although the overall concentrations of the combined pools of these cofactors are highly regulated (remaining virtually constant such that individual reactions have little effect), changes in their individual concentrations are known to play important roles in the control of other metabolic functions such as glycolysis (*27*). Therefore, the inclusion of these metabolites was considered important to capture the entire range of potential links within the network.

The functional roles that motifs might play in metabolic networks remain unclear. However, recent comparative studies have found that similarities in the proportions of enzyme classes between species were related to structural features of the motifs present (*7*). Comparisons of motif distributions across different organelles within a cell also showed distinct differences that are thought to relate to the specific metabolic functions of each compartment (*7*). These differences will directly affect the types of motif clustering that are possible, and so the highly limited forms that we find for FFLs are likely mirrored for other motifs that are prevalent. To test this further, we selected and analyzed an additional set of five metabolic networks from bacteria and archaea spanning a broad range of classes for both FFL and FBL motifs (fig. S4). These again showed a strong bias for type 6 FFL clusters and type 2 FBL clusters. Furthermore, for both types of motif, increases in overall motif clustering saw stronger biases for the specific clustering types observed.

Highly constrained FFL clustering types were also found for the transcription factor networks (fig. S5) where two types of FFL clustering, types 6 and 12, make up 80 to 90% of the total (Fig. 2C). In type 6 FFL clusters, a single master regulator separately regulates two pairs of genes, where, for each pair, one gene is a transcription factor regulating the other. Type 12 FFL clusters also have a single transcription factor regulating others in the cluster, but in this case, the regulator and its target coregulate multiple target genes. Type 12 FFL clusters enable closer coordination between the expression patterns of groups of genes and have been shown to enable temporal regulation of target genes (for example, ordered activation) (*15*).

A striking feature of these motif clusters is the simple hierarchical structure known to be present in these types of network (*28*, *29*). This is most evident in the *E. coli* network where the seven separated components all share highly similar architectures (fig. S5A). In this network, we tend to find that intermediate nodes connect to only a few targets (outputs), as shown in the cluster regulated by cAMP (adenosine 3′,5′-monophosphate) receptor protein (CRP). In this case, the large number of separated intermediaries relates to the control of different metabolic processes, allowing for a signal from the master regulator (CRP) to be adjusted for a specific process’ needs. This separation also accounts for the large numbers of type 6 FFL cluster in the motif clustering type distributions (Fig. 2C). Moreover, the highly specific types of FFL clustering displayed suggest that these structures exhibit important functional benefits or that other possible types of clustering see strong negative selection. The former is supported by dynamical analyses of the potential functions that such structures enable (*3*, *8*, *9*, *11*).

The *S. cerevisiae* FFL clusters also exhibit a hierarchical structure but of a far more integrated form (that is, fewer inputs and intermediaries control many outputs; fig. S5B) (*30*). Lee *et al.* (*31*) also reported this feature, finding that the number of promoter regions bound by a regulator ranged from 0 to 181 with an average of 38 interactions per regulator, with many interactions in the same functional category. This is clearly illustrated for the large motif cluster controlled by GLN3 and DAL80 that contains 17 output genes, all regulated by these same two inputs (fig. S5B). This more integrated architecture leads to an increase in the overall FFL clustering shown by the associated *z* score (table S2) and helps explain the elevated proportion of type 12 FFL cluster where a single input and intermediate are connected to large numbers of outputs (Fig. 2C).

Unlike the *E. coli* network, the *S. cerevisiae* FFLs display greater variation in their types of clustering. Diversification of motif clusters related to specific cellular processes leads to a more complex structure where multiple inputs are integrated to regulate target genes for different cellular functions. This integrative structure is evident in the large motif cluster that contains TUP1 as a central input (fig. S5B). Broadly, this cluster breaks up into three main parts: mating type switching, meiosis, and biosynthesis and respiration. Although each of these cellular processes is controlled in isolation by several independent inputs, more complex phenotypes such as the switching of mating type require the coordination of many of these processes in unison. The TUP1 gene fulfills this role and has experimentally been shown to act as a global transcriptional repressor (*32*).

One of the most widely studied examples of an ecological network is the Little Rock Lake food web (*23*). Nodes represent species, and edges denote the consumption of one species by another. Most of the FFL clusters in this network (61%) are of types 1 and 2 (Fig. 2C and fig. S6). In these clusters, one node is a predator that consumes all or most of the other species in the cluster. The prevalence of these structures reflects the fact that most of the species in the lake are consumed by other species across trophic levels. Only a few top predators exist, with most of these acting as omnivores.

The role of omnivory in food webs is still contested (*33*–*37*). Initial theoretical studies found that its presence under equilibrium conditions can destabilize food webs (*33*). However, more detailed collection of species interactions has shown it to be a common property of many types of food web (*35*). More recently, nonequilibrium studies of these networks (*36*, *37*) and consideration of potential adaptive mechanisms (*36*) have revealed that omnivory can help improve system stability and damp potential chaotic dynamics. The FFL motif captures this key relationship, and motif clustering captures the higher-order forms it can take. The prevalence of this and derived structures in the food web is evident with FFL clusters comprising 54% of all nodes and 38% of all edges across the entire network (table S3). Detailed investigation of other FFL clustering types also showed a decreasing number of longer-range predator-prey interactions across more distant trophic levels. We found two- and three-level predation to be most common (FFL clustering types 1 and 2), whereas four-level relationships were very rare. This pattern is due to the limited number of trophic levels present in this food web. Furthermore, we find it rare for multiple top-level predators to share the same low-level but alternative intermediate-level prey. This is due to adaptations to consume one form of prey likely having similar benefits on potential higher-level prey.

In the *C. elegans* neural network, 58% of the FFL clusters are also of type 1 or 2 (Fig. 2C and fig. S7). In these networks, the nodes are neurons and the edges are synapses. Because this is a neural network, type 1 and type 2 FFL clusters are where a node receives information from all or most of the other nodes in the cluster. This is consistent with a highly integrated structure, with many nodes receiving and integrating information from multiple sources. Furthermore, theoretical studies of the dynamics of FFL structures in neural networks have shown their potential role in local stability (*38*), as well as permitting input events that do not occur exactly simultaneously to trigger a response through the use of the intermediate node as memory (*15*). Apart from these FFL clustering types, others where one and two nodes are shared (types 2 to 6 and 7 to 12) have similar proportions. Kashtan *et al.* (*15*) showed that generalized motifs maintain the same function as their underlying motif over a broader range of nodes. Therefore, combining the many functions that motifs have been shown to exhibit with the ability for neural networks to tune available connection strengths through synaptic plasticity makes the wide range of FFL clustering types capable of many different forms of information processing.

#### Motif clustering types in engineered and social networks

In contrast, engineered and social networks were dominated by alternative types of FFL clustering. The Wikipedia vote (fig. S8) and air traffic control networks (fig. S9) saw type 6 FFL clusters highly expressed, at 39 and 32%, respectively. For the voting network, elections arise when a user requests to become an administrator. Other Wikipedia users can then vote on who they want to promote. Nodes in the network correspond to users, and an edge signifies one user voting for another. The strong bias for type 6 FFL clustering reflects the fact that the preference of a voter is likely to match the candidates they vote for. Therefore, in future elections, although both a user and a previous candidate may have no direct social ties, their similar preferences will make it more likely for them both to vote for the same candidate. This embeds type 6 FFL clusters within the network. Such relationships arise from the underlying homophily present in virtually all social networks (*39*–*41*), which has been shown to emerge under many different conditions (*42*).

Similarly, for the air traffic control network, a major influence on the structure is a requirement for robust paths to multiple destinations while taking into account geographical limitations. In this network, nodes are airports and edges are recommended routes. FFL clustering types 5 and 6, both highly expressed, embody a function where a single input spreads out to many intermediate and output nodes. This suggests that recommended routes attempt to reduce the local burden on specific airports, sharing traffic that may accumulate during difficulties (for example, weather disruptions).

The Gnutella network (fig. S10) saw a large proportion of FFL clustering types 4 and 12, making up 33 and 18% of the FFL clusters, respectively (Fig. 2C). These FFL clusters include only a single intermediate node. In this network, nodes represent computers (clients or servers) and edges denote the transmission paths between them. Gnutella is a decentralized file-sharing protocol. When a new computer connects to this network and requests a file, it first searches for a local “ultrapeer” server. These are special nodes that act as a high-speed backbone for the network and are purposefully spread out to improve communication efficiency. Once a client is connected, the request is processed and forwarded to the appropriate target server. The clients can then directly connect, leading to an FFL transmission structure being generated in the network. FFL clustering types 4 and 12 both capture this process whereby many clients (inputs) connect to target servers (outputs), using a single ultrapeer server (intermediate).

Finally, the EU email network (fig. S11) exhibits larger proportions of type 7 FFL clustering related to multiple inputs and a shared intermediate and output and type 9 FFL clustering related to a three-level hierarchy where two intermediate nodes are also connected (Fig. 2C). The majority of email within an organization will take place via an organizational hierarchy, and these particular motifs are a likely consequence. One interesting aspect is a lack of other hierarchical motifs being expressed. This reflects a segregated structure where it is uncommon for high-level managers to directly interact with those more than two levels below. It would also account for type 9 FFL clusters having the largest overall expression of 22% because this type separates the main input from the output through an intermediate layer.

### Robustness of motif clustering to edge removal

To ensure that the specific types of FFL clustering were a robust feature of the networks, we randomly removed 1 to 50% of edges from each network and calculated average FFL clustering type distributions from 500 trials at each percentage. The Wikipedia vote and EU email networks were omitted because of their size and density, leading to unfeasible execution times for a sufficient number of samples. In terms of specific motifs, we would expect random edge removal to affect their counts in proportion to the number of edges they contain. Therefore, FFL clusters consisting of a single shared node (six edges) would be expected to see a greater impact than FFL clusters sharing two nodes (five edges).

We found that, for most networks, the FFL clustering type distributions are highly robust to random edge removal (fig. S12). Some networks, such as the Little Rock Lake food web and air traffic control networks, show virtually no change in their FFL clustering type distributions even after 50% of all edges have been removed. The robustness of this feature can be accounted for by the high FFL clustering found in all the networks that we considered and the majority of this being in the form of a single shared node (that is, FFL clustering types 1 to 6). In this case, removal of an edge results in the elimination of a single FFL, localizing the effect on the overall motif clustering. To help illustrate why this is the case, consider a network that contains a single central node that is shared by all FFLs in the network. No matter which edge is chosen, only a single FFL will be affected. Similarly, for the case where FFLs are clustered around a pair of nodes, there is the danger that removal of this central edge will result in all FFLs being lost. However, as the clustering increases, the probability of picking this edge rapidly diminishes, leading to a structure where random edge removal is again only likely to affect a single motif. Therefore, if clustering is high and most motifs are clustered through a single shared node, structural perturbations become localized to the motif in which they occur.

The networks that see the most sensitivity to edge removal were those of metabolism. Although small amounts of edge removal up to 15% lead to minor changes in the overall proportions of FFL clustering types, as further edges were removed, a rapid breakdown of these features was seen. At 50% edge removal, we find that FFL clustering types 1 to 6 and 7 to 12 have become heavily homogenized for both metabolic networks. A possible reason for this breakdown may relate to the highly optimized nature of metabolism. Of all the distributions that we analyzed, metabolic networks displayed the most highly organized form, with nearly 80% of all FFL clustering being of a single type. Further analysis revealed that this is due to a highly clustered region containing a few central nodes (figs. S1 and S2). This extreme level of clustering causes most of the edges in the network to be concentrated within a few FFL clusters and increases the probability that an edge removal will affect these structures. Unlike other networks where similar features are found (for example, Little Rock Lake food web and *C. elegans* neural network), for the metabolic networks, the reduced number of central nodes leads to an increased density, with every FFL being connected to a far greater number of others. Therefore, the removal of any of these FFLs has a larger impact on the types of FFL clustering present in the network.

### Information flow through the motif clusters

**Fig. 3:**
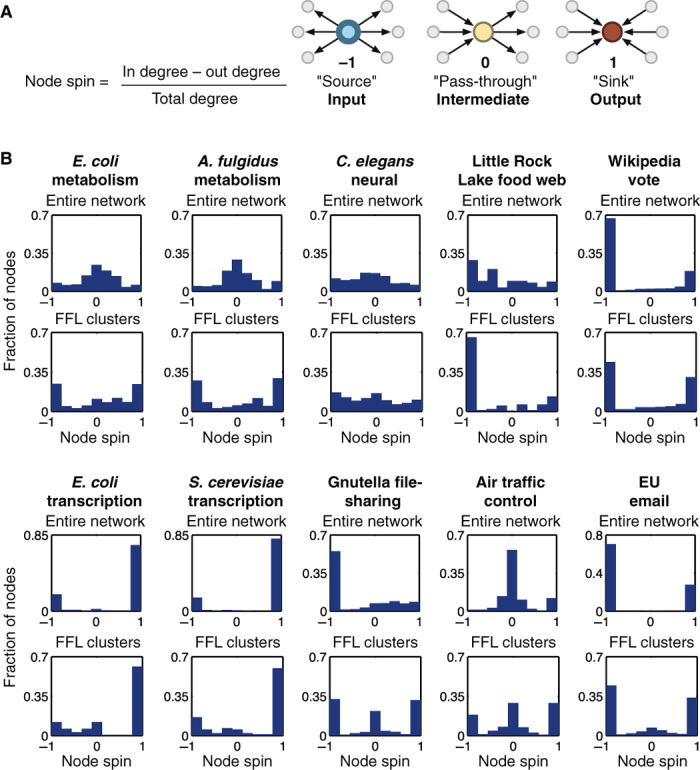
Node spin distributions. (**A**) Node spin is defined as the normalized difference between the in degree and out degree of a node, classifying its role as producing (source), receiving (sink), or relaying (pass-through) information. (**B**) Node spin distributions for the entire real-world networks (top) and the extracted FFL motif clusters (bottom).

Our motif clustering results suggested that some networks have very strong prevalence for certain types of information flow (according to our results, above 70% of the FFL clusters in a network might be of the same type and, hence, produce similar types of information flow). Our classification of motif clustering types identified nodes as input, output, or intermediate, but the intermediate class encompasses nodes with a range of very different characteristics. To further dissect information flow within the FFL clusters, we applied an approach similar to that of Ma’ayan *et al.* (*10*) and used the notion of node spin to classify the extent of each node as a producer, receiver, or relayer of information (Fig. 3A). We compared the node spins of whole networks to those of their FFL clusters (Fig. 3B and table S3). In several cases, the extracted FFLs and the whole network have similar profiles, but there are examples where the FFLs contribute very differently to information flow from the whole network (for example, metabolism).

The transcription networks have very polarized node spin distributions biased toward inputs and outputs, which were similar for the entire network and extracted FFLs (Fig. 3B). Analysis of the FFL clusters revealed an output-centric structure, with approximately 60% of nodes acting as outputs. The remaining nodes have an input role, although only a small percentage (12% for *E. coli* and 16% for *S. cerevisiae*) act solely as inputs. Closer inspection finds that nodes acting solely as inputs are master regulators. For example, in the *E. coli* network, we find the input node CRP, which has been found to regulate more than 180 genes related to catabolism of secondary carbon sources (*43*), as well as other cellular processes such as biofilm formation, virulence, and nitrogen assimilation to name but a few. Another input node, FNR (fumarate-nitrate reduction regulatory protein), regulates hundreds of genes to control the transition from aerobic to anaerobic growth (*44*), as well as many other cellular functions. The overall structure of the FFL clusters permits incoming information to be integrated through relatively few intermediate nodes before being relayed to large numbers of outputs.

In contrast, the metabolic networks displayed a bias for intermediate node spins in the entire network and for input and output node spins in the FFL clusters (Fig. 3B). These differences could be explained by large portions of the entire metabolic networks consisting of sequential transformations where the product of one reaction becomes the substrate for another. These sequences of reactions lead to nodes that are balanced (that is, having similar numbers of inputs and outputs) and result in the node spin distribution peaking around zero. Conversely, the network of the extracted FFLs has a highly interconnected structure (figs. S1 and S2). This is due to commonly used cofactors (for example, ATP), which bind the network together. When the product of one reaction is the substrate to another, and when both also require one of these cofactors, an FFL is created with all reactions where the cofactor is a product (for example, ATP phosphoribosyltransferase). The cofactor-producing reactions act as inputs, and because many of the products from these final reactions will feed into other reactions that do not require the same cofactor (and so are likely to not be included in the extracted FFL networks), these reactions become outputs and lead to the strong bias for input and output node spins (Fig. 3B).

The incredibly flat distribution of node spins for the *C. elegans* neural network shows that routes for information flow are evenly distributed with a broad range of input/output ratios. It has been shown that FFLs in these networks can act as decision points, triggering when sufficient numbers of inputs are activated within a short time window (*15*). By having a wide range of potential input/output ratios present in the network, and with the previously highlighted ability of these networks to dynamically adapt through synaptic plasticity, they are flexible to a broad set of alternative functions.

A striking feature of the nonbiological networks is the convergent node spin profiles of FFL clusters in the Gnutella file sharing, air traffic control, and EU email networks. Although these are taken from very different domains, this convergence reflects the precisely defined roles of components in these engineered networks, where the vast majority of nodes are solely input, solely output, or solely intermediate. This segregation is generally due to the functional modularity that most engineered systems have. This enables the systems to predictably grow in complexity, with specific tasks encapsulated within particular elements of the system. For example, in the case of the Gnutella file-sharing network, inputs and outputs represent the end users and information providers, whereas the task of information transmission (intermediate spins) is performed by a specialized set of ultrapeers whose major role is the relay of information. In addition, the transmission of information (Gnutella and EU email) or even planes (air traffic control) relies on the ability of these rely points to not act as potential bottlenecks. This would account for the large proportion of near-zero node spins where a balance of inputs to outputs is present.

Similarly, the Wikipedia vote network displayed a strong convergence to an input- and output-oriented architecture, with a smaller fraction of nodes having an output bias (small ramp in the distributions). This clearly captures the bipartite nature of the voting system, containing members that can be classified as either voters (inputs) or candidates (outputs). Furthermore, as a global feature, the extracted FFL clusters display a similar distribution to the entire network, which accounts for a large proportion of its structure (54% of nodes and 85% of edges; table S3).

### Comparison to random graph models

**Fig. 4:**
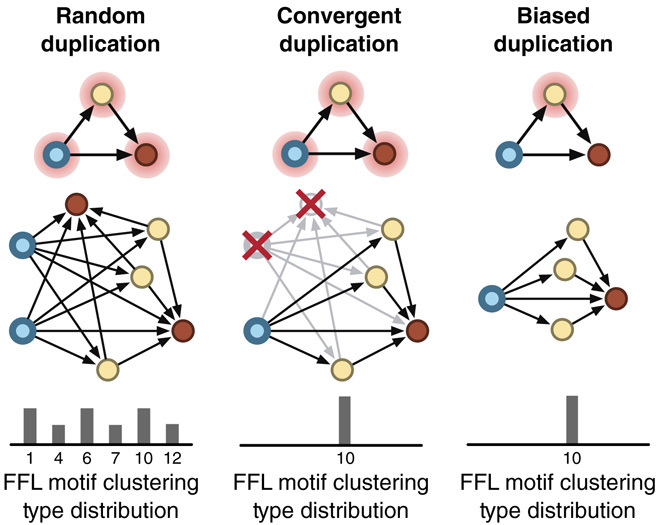
Routes to biased motif clustering distributions for a duplication-based growth process. Random duplication leads to a broad distribution of specific motif clustering types. In contrast, biased distributions exhibiting a single type of clustering can occur through convergent duplication where all nodes remain equally likely to be duplicated, but post-duplication processes (such as natural selection) remove nonfunctional or deleterious events. Alternatively, the duplication event itself could be biased, with some types of node having higher probability than others. This bias could be due to functional limitations in the system (for example, physical cabling restrictions in communication networks) or an intrinsic feature of the system itself (for example, a mutational bias in genetic systems).

To test whether the types of motif clustering observed might be the results of random evolutionary processes, we considered two random network models. The first was an Erdős-Rényi model where every directed edge had a fixed independent probability. The second was a node duplication model where the network was initiated with a template motif and then nodes were randomly chosen and duplicated, including all associated edges. These displayed highly structured motif clustering type distributions with clear signatures in the factions of particular motif clustering types (Fig. 2C; see text S3, figs. S13 to S15, and table S4 for an analytical derivation of the expected motif clustering type distributions for both random models). In all cases, the real-world networks showed biases in the clustering type distributions that could not be produced by random wiring (Erdős-Rényi) or unbiased node duplication (Fig. 2C). As we would expect, this suggests that more complex evolutionary mechanisms may be at work. For example, biased node duplications or strong selective pressures allow only limited forms of motif clustering to emerge (Fig. 4).

### Role of evolution by duplication and divergence

For many complex systems, duplication and divergence form an important part of their evolution and are linked to the formation of highly overlapping motif clusters. Duplication of any node within a motif will lead to a new motif of the same type being created that is fully clustered with the original. This proliferation of motifs and clustering provides a direct link between an evolutionary process and the motif clustering properties of a network. Furthermore, duplication-based evolutionary processes leave a mark on the systems they shape. This is evident from the random node duplication model, which exhibits increased numbers of FFL clustering types 7, 10, and 12 (Fig. 2C). Each of these is directly related to duplication of an input, intermediate, or output node from the original FFL. For networks where duplication is known to occur, it is possible to use this feature to interpret how these processes contribute to the evolution of the system.

Although a random duplication process will lead to equal increases in FFL clustering types 7, 10, and 12, this is not always the case in a real-world system. Selective pressures restrict the types of duplication maintained because of differences in the fitness benefit that they provide. Furthermore, the processes that lead to duplication may be biased, leading to different amounts of each motif clustering type (Fig. 4). By making use of this information, we are provided with an opportunity to measure selective pressures or mechanistic biases from static network data.

Having found that biases in motif clustering type distributions may offer a powerful means to detect remnants of an evolutionary process, we attempted to search for signs that duplication and divergence had played a role in the real-world networks. We searched for biases in FFL clustering types 7, 10, and 12, related to duplication of an input, intermediate, and output node, respectively. We focused on networks where duplication-based evolutionary processes are thought to be present (that is, Little Rock Lake food web, metabolism, and transcriptional regulatory networks). In food webs, duplication and divergence form an important aspect of speciation. In particular, speciation is thought to take place through several gradual processes that see a single population diverge because of genetic polymorphism (sympatric), barrier formation between separated groups (allopatric), the creation of an isolated subpopulation where gene flow is restricted (peripatric), or the creation of a partially separated subpopulation where localized evolution eventually reduces the fitness of reproductive events between the two groups (parapatric). Duplication and divergence have also been shown to be a primary mechanism for the evolution of genetic systems (*45*), covering both metabolic and transcriptional regulatory networks.

To test whether duplication had left a mark on the FFL clusters, we analyzed the FFL clustering type distributions (Fig. 2C). For the Little Rock Lake food web, we found increased proportions of all FFL clustering types related to single node duplication. Furthermore, a large bias was seen toward type 7 FFL clusters. This is related to the duplication of an input (producer). With no known mechanistic biases during speciation, this would suggest that low-level prey are under increased pressure to diversify so as to evade generalist predators.

For the metabolic networks, we again find a clear signature of duplication-based events, with FFL clustering types 7, 10, and 12 all expressed in higher quantities than other types sharing two nodes (Fig. 2C). In contrast to the Little Rock Lake food web, metabolic networks saw increased proportions of output-based nodes in the FFLs (type 12 FFL clusters). Considering that genetic duplication-based events are thought to be, for the most part, random, the reduced selective pressures for this type of duplication event may relate to the fact that metabolic networks are essential to life and are therefore highly optimized and controlled. Changes to this process are unlikely to be beneficial to the overall fitness of an organism, and so it is reasonable that biases may favor those duplication events that have reduced overall effect. In the case of an FFL, the input node has the greatest impact, followed by the intermediate node and, finally, the output. This would predict a bias toward output nodes, as seen in the metabolic networks. In addition, diversification of outputs could also have a positive effect, with diverse metabolic products providing a larger variety of materials for a cell to build from.

The transcriptional regulatory networks also exhibit a strongly biased distribution toward duplication of output nodes but display virtually no duplication of input or intermediate nodes (Fig. 2C). Again, as in the metabolic network, this may relate to limiting the impact of deleterious changes. However, the lack of inputs and intermediates but large numbers of outputs also suggests that FFLs with duplicated outputs form structures with beneficial functions that are positively selected for. This is supported by studies of bifan networks, which are closely related to output-focused FFL clusters. Such structures have been shown to be capable of generating a wide range of useful behaviors (*13*).

### Testing for evolution by node duplication

Because of the strength of the bias in the transcriptional regulatory networks and the ability to capture evolutionary relationships through DNA sequence data, we attempted to verify whether duplication had played a role in the creation of these FFL clusters. Concentrating on the *E. coli* transcriptional regulatory network, we separated each group of output nodes (target operons) into candidate sets that shared the same input and intermediate node (that is, they were members of the same FFL motif cluster). This resulted in nine candidate operon sets (table S5). To verify whether duplication had taken place, it was necessary to compare sequences that make up each of these operons and see whether similarities are found within the same candidate set. A major difficulty when comparing genetic sequences is the continual evolution they undergo. Although sometimes this will relate to the evolution of a new functional protein, the redundancy in the genetic code allows for neutral evolution to also occur. Neutral evolution relates to genetic changes to an individual that have no measurable effect on their fitness or such a small effect as to not have an impact on reproductive success. This random drift allows for two identical genes generated by a duplication event to slowly diverge over time. This makes direct comparison of their DNA sequences difficult.

An alternative approach is to consider structural characteristics of the proteins that a sequence encodes. Protein structure is more highly conserved over time, and so structural classifications enable the identification of more distant relationships (*46*). We gathered structural domain assignments from the SUPERFAMILY database (*47*) for each of the proteins coded for by our candidate operons, and because operons can encode many proteins, this leads to multiple assignments for each operon (table S5). This revealed relatively few duplication events within candidate sets (that is, two different operons from the same candidate set having the same structural domain assignments). However, of those that we did detect, most occurred in sets containing only a few operons (candidate sets 1, 4, 5, and 9; table S5). This reduces the chance they could have occurred by other means and supports duplication playing a role in the creation of these motif clusters.

For the other candidate sets, strong selective pressures and an ability to rewire regulatory connection would be expected for the FFL clusters to form. This is supported by the flexible nature of regulatory interactions. Teichmann and Babu (*48*) have shown in genome-wide studies of network evolution in *E. coli* and *S. cerevisiae* that duplication of a target gene and its regulatory element accounts for only 10% of interactions. The majority of interactions, approximately 90%, instead form because of duplication of the transcription factors or regulatory components, enabling new regulatory interactions to be easily formed. Therefore, a combination of both duplication and strong selective pressures likely underpins the FFL clustering that we observe in the transcriptional networks.

### Identifying functionally important nodes

**Fig. 5:**
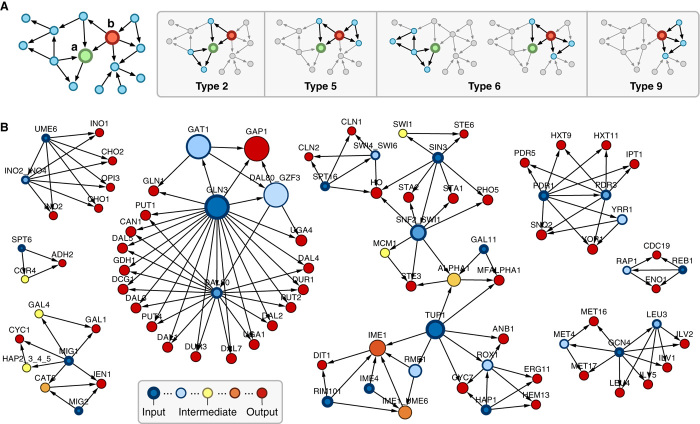
Overview of the MCD measure. (**A**) Example where two nodes have been selected (left) and highlighted when they are a member of a clustered motif pair (right). The MCD is given by the number of different motif clustering types a node is a member of. Therefore, although both are a member of four unique motif pairs, “a” has an MCD value of 3, whereas “b” has a value of 4, because of two motif pairs that include “a” having the same motif clustering type 6. (**B**) FFL motifs extracted from the *S. cerevisiae* network. Node size corresponds to MCD, and color represents the node spin. Clusters around the TUP1 gene correspond to (top) mating type switching, (bottom left) meiosis, and (bottom right) biosynthesis and respiration.

Having tested for evidence that groups of nodes might duplicate, preserving their connections, we developed a way to identify nodes whose connections must have arisen in other ways. In complex networks, nodes with critical functions tend to act across or coordinate many different processes. Most measures of node importance in a network rely on simple structural features (for example, degree) or bottlenecks in the routes between nodes (for example, betweenness). However, these features do not fully capture a coordinating behavior. Because motif clusters are a structure connecting numerous parts, it is easy for them to be lost during evolution unless they are actively selected for and maintained. This results in separate clusters often being linked to core functionalities (Fig. 5). Although nodes within a cluster may have a high connectivity (for example, DAL80 in Fig. 5B), because the interactions are limited to within the motif cluster, they are unlikely to play a broader role in coordination of many functions across the system. Therefore, we hypothesized that nodes spanning motif clusters of many different types might better capture those playing a key role in coordination and, thus, are important to the system. We defined a motif clustering diversity (MCD) measure, which was calculated as the number of different motif clustering types that a node takes part in (Fig. 5A and Materials and Methods). As a test, we applied this measure to the *S. cerevisiae* transcription network to see whether functionally important nodes could be found (Fig. 5B). The nodes with the highest MCD were TUP1, GLN3, GAP1, GAT1, and DAL80. As mentioned earlier, TUP1 is a global corepressor of transcription (*32*) that switches off well over a hundred genes in diverse signaling pathways. GLN3, GAP1, GAT1, and DAL80 together directly or indirectly regulate hundreds of genes to optimize nutrition (*49*).

**Fig. 6:**
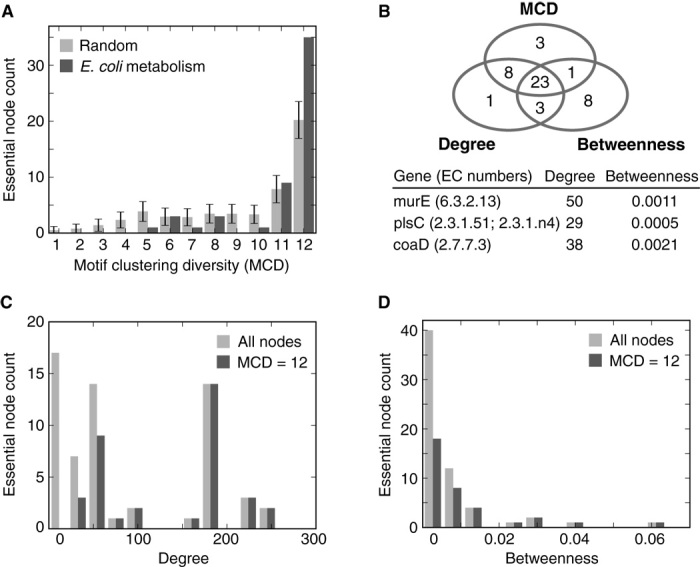
Capturing essential nodes of the *E. coli* metabolism network using MCD. (**A**) Comparison of the number of essential nodes selected for each MCD value versus a random selection model that accounts for nonuniform group sizes. (**B**) Venn diagram of essential enzymes selected for the top 156 nodes with greatest importance in regard to our measure (that is, MCD = 12). We compared MCD, degree, and betweenness for all nodes in this set. The three nodes exclusively captured by the MCD are displayed in the table below. (**C** and **D**) Comparison of all essential nodes in the metabolic network to all essential nodes with an MCD = 12 and the associated (C) degree and (D) betweenness.

To test this hypothesis further, we used experimental data on essential genes in *E. coli* (*50*) and assessed whether high MCD values of enzymes in the metabolic network were a good predictor of their essentiality. The MCD was calculated for every essential node in the network, and we found that the highest MCD value of 12 was significantly enriched (*z* score = 4.6, adjusting for different group sizes at each MCD value; Fig. 6A). We also found that FFL motif clusters were significantly enriched with essential nodes, suggesting that these particular motifs play an important functional role. We compared the accuracy of our predictions using MCD to those of other standard network measures of node importance, specifically node degree and betweenness centrality (Fig. 6). We analyzed the hit rate of essential nodes for the top 156 nodes with the highest degree, betweenness, and MCD. This group size was chosen because it captured the full set of nodes with MCD = 12 (covering both essential and nonessential genes) and resulted in selecting nodes with a betweenness ≥0.0029 and degree ≥50. Although MCD displayed excellent performance, a striking difference in its predictions to the other measures is shown in Fig. 6. The MCD predicted nodes exhibiting a broad range of degree and betweenness values and resulted in three essential nodes (~10%) being uniquely identified by the MCD measure (Fig. 6B). This suggests that MCD and motif clustering more generally provide a complementary approach that can suggest functionally important targets missed by other methods.

## DISCUSSION

We have explored the way in which motifs can act as building blocks for complex networks and attempted to better understand their connection architecture across several real-world systems. Our analysis revealed that aggregation of FFLs into clusters is significant. Detailed analysis showed that these motifs can potentially become connected in a large number of different ways, but only a few are in fact used within the real-world networks. In some cases, just one or two types of clustering dominate. This suggests that limited rules may underlie the ways that motifs can be successfully pieced together to generate larger structures with functionally useful dynamics. In addition, biases in the motif clustering types were shown to capture key features of underlying evolutionary processes, such as duplication and divergence, in addition to selective pressures placed upon these processes. Motif clustering offers a glimpse at functionally important structures and the evolutionary process by which they arise. Furthermore, the important role that motif clustering plays was illustrated in terms of the essential nodes within the metabolic network of *E. coli*. The MCD measure was shown to predict essential nodes with comparable performance to existing approaches while also highlighting other nodes that were missed.

There has been much debate over the functional role of network motifs in complex networks (*2*–*7*, *9*–*11*, *14*). Much of the difficulty in answering this question stems from our often limited understanding as to what constitutes a “useful” function in the context of the system being studied. For example, transcriptional networks clearly regulate gene expression to allow for adaptation and survival of a cell, but a useful function for a particular cell will depend on the dynamic environment in which it lives, as well as the effect that each gene has on phenotype. In most cases, neither of these aspects is fully known, making it difficult, if not impossible, to resolve a motif’s complete functional role.

An alternative way of assessing whether a motif has a useful function is to see whether there is evolutionary selection for it. This can be done by comparing the observed networks to those generated by a null network model. These null models can be purely structural, maintaining the statistics of a set of network features (*51*), or can be derived from simulations of the system itself (*52*, *53*). In this case, although the precise function of the motif may not be known, deviations from null models provide a strong indication of those structures important to the system’s overall function. Motifs are generally found this way (*2*), but as mentioned previously, studying the dynamics of these motifs in isolation may not be relevant to their context within a larger network (*14*). Our motif clustering method helps to alleviate this issue by extracting common contexts (that is, motif clusters) that better capture the actual connection structures that individual motifs experience. This will support future detailed investigations into the dynamic properties such structures exhibit.

The focus of this work has been on motif clustering across entire networks. Although this has helped identify system-wide architectural principles, many networks are composed of loosely connected communities that often perform distinct functional roles. An intriguing future direction would be to apply these methods at the multiple resolutions present within a network. This will enable differences in the motif clustering rules to be captured and further refine our understanding of how and why certain types of connection are favored over others.

## MATERIALS AND METHODS

### Motif clustering coefficient

The motif clustering coefficient *M*_c_ attempts to capture the overlap between motifs in terms of the number of shared nodes between all pairs of motif of interest within a network. To calculate the motif clustering coefficient for a network G=(V,E), we considered a set of motif types $M=\{M_{1},M_{2},\ldots \}$, where each $M_{i}=(V_{i}^{M},E_{i}^{M}),\forall i$ defines a network that captures the motif structure. We also ensured that *M*_*i*_ ⊈ *M*_*j*_, ∀ *i*, *j*, where *i* ≠ *j* such that no motif is a subisomorphism of any other. For each motif type, $M_{i}\in M$, we searched for instances (subisomorphisms) in G and, for each motif occurrence, added the associated nodes for the motif from G to the set of found motifs *F* = {*f*_1_, *f*_2_, …, *f*_*n*_}, where $f_{j}\subseteq V,\forall j$. Therefore, *F* contains sets of nodes from G where each set defines one of the motif types in M. This set was calculated using the VF2 algorithm (*54*). The motif clustering coefficient is then given by

$$M_{c}=\frac{S}{T}$$ (1)

where *S* is the total number of shared nodes between all pairs of found motif in *F*, and *T* is the total possible number of shared nodes had all pairs of these motifs been fully clustered (sharing the maximum possible number of nodes). These were calculated using

$$S=\sum_{i=1}^{n}\sum_{j=i+1}^{n}|f_{i}\cap f_{j}|$$ (2)

$$T=\sum_{i=1}^{n}\sum_{j=i+1}^{n}[\text{min}(|f_{i}|,|f_{j}|)-1]$$ (3)

where *n* is the number of motifs in the network, *f*_*i*_ is the set of nodes that make up motif *i*, and |⋅| denotes set cardinality. In the case where only a single type of motif is considered (that is, M={M}), then $T=(\frac{n}{2})(|M|-1)$, where |*M*| is the number of nodes in motif *M* and $\left(\frac{n}{2}\right)$ is the binomial coefficient specifying the number of ways of choosing two elements from a set of size *n* without taking order into account.

For cases where M contains several motifs, there are two further types of motif clustering that we can consider: homologous and heterologous. These were obtained by partitioning the set of found motifs (*F*) as $F=F_{1}\cup F_{2}\cup \ldots \cup F_{\left|M\right|}$, where each subset *F*_*i*_ = {*f*_*i*1_, *f*_*i*2_, …} contained only those nodes that define motifs in the network G of type *M*_*i*_. Next, we generalized *S* and *T* to consider the number of shared nodes between two specific types of motif

$$S_{ij}=\sum_{a=1}^{\left|F_{i}\right|}\sum_{b=a+1}^{\left|F_{j}\right|}|f_{ia}\cap f_{jb}|$$ (4)

$$T_{ij}=\sum_{a=1}^{\left|F_{i}\right|}\sum_{b=a+1}^{\left|F_{j}\right|}\;\text{min}(|f_{ia}|,|f_{jb}|)-1$$ (5)

where *S*_*ij*_ is the number of shared nodes between motifs of type *M*_*i*_ and *M*_*j*_ and *T*_*ij*_ is the total possible number of shared nodes between the sets of motifs *M*_*i*_ and *M*_*j*_. Then, we were able to consider the clustering between motifs of the same type and define homologous motif clustering as

$$M_{c}^{+}=\frac{\sum_{i=1}^{\left|M\right|}S_{ii}}{\sum_{j=1}^{\left|M\right|}T_{jj}}$$ (6)

Conversely, by considering the clustering between different types of motif, heterologous motif clustering can be defined as

$$M_{c}^{\pm }=\frac{\sum_{i=1}^{|M|-1}\sum_{j=i+1}^{\left|M\right|}S_{ij}}{\sum_{p=1}^{|M|-1}\sum_{q=p+1}^{\left|M\right|}T_{pq}}$$ (7)

An example for a simple network can be found in text S2.

To calculate the statistical significance of a motif clustering coefficient, we used rejection-based sampling where random networks were generated maintaining the same number of nodes, edges, and motifs as the original. This was performed by starting with an empty network containing the same number of nodes as the original. Motifs were then individually placed at random until the same number of motifs was present. Finally, any outstanding edges were randomly placed to ensure that the same number of edges was also maintained. If at any point during this process the number of motifs or edges exceeded those found in the original network, the randomized network was rejected and the process was restarted. The motif clustering coefficient was calculated for each random network, and a comparison was made to the original network using a standard *z* score.

### Motif clustering type distributions

To calculate the motif clustering type distribution for a particular set of motif types $M=\{M_{1},M_{2},\ldots \}$, we first generated a set of all possible motif clustering types $C=\{c_{1},c_{2},\ldots \}$, where each member $c_{i}=(V_{i}^{c},E_{i}^{c}),\forall i$ is a network. Specifically, C contains networks representing all the unique ways that two motifs from M can become clustered sharing at least one node in common. To do this, we enumerated over all possible overlaps between all motifs in M, including the case where a particular motif is clustered with itself (for example, if M contained a single FFL motif, then the set of motif clustering types *C* would contain all the networks shown in Fig. 2A). When generating this set, it is important to ensure that all clustering types are unique by checking that any newly generated candidates are not isomorphic to an existing member of C. This ensures that symmetries in a motif do not lead to multiple clustering types that have the same network structure. Once the set C of motif clustering types has been generated, we searched for all instances of the motif types in M within the network of interest G using the same approach as for the motif clustering coefficient. For all pairs of motif found, we extracted them from the original network and compared the resultant subgraph to the set of motif clustering types in C. Cases where motifs do not share any nodes are neglected. We counted the occurrences of each motif clustering type and normalized this by the sum total of all motif clustering type counts. As with the motif clustering coefficient, this measure is easily adapted to homologous and heterologous cases by generating motif clustering types C that only enumerate clustering between the same or different types of motif in M, respectively.

### Motif clustering diversity

The MCD of a node was calculated by extracting all motifs of interest that contain the selected node as a member. We then considered all pairs of these motifs and, for each, classified their type in C using the same method as described for the motif clustering type distributions. The MCD measure was then given by the number of different types of motif clustering in C that the node is a member of (Fig. 5A).

### Motif clustering analysis

To test for the significance of FFL motifs in the real-world data sets, 10,000 randomized networks were generated for comparison using the method described by Wernicke (*51*), which maintains the number of nodes, edges, and degree sequence. The EU email network and Wikipedia vote networks were omitted because of their size and density, making sufficient sampling of randomized networks unfeasible. Specifically, the number of edges in these networks made randomized placement of motifs and edges without increasing the overall number of motifs difficult, leading to a high rejection rate of candidate networks. To test for motif clustering between FFLs, we again used 10,000 randomized networks to assess the significance of the motif clustering, but this time maintained the number of nodes, edges, and motif count.

### Classifying essential nodes

We tested whether motif clustering allowed for the improved prediction of essential nodes in the metabolic network of *E. coli* derived by Chang *et al.* (*24*) by combining information on essential genes gathered by Gerdes *et al.* (*50*). The metabolic network consisted of nodes that represented enzymes with an Enzyme Commission (EC) classification number as a label. Therefore, to classify particular nodes as essential, EC numbers were extracted for each essential gene in the study of Gerdes *et al.* (*50*). This was performed using the UniProt representational state transfer web interface. Queries were executed for each essential gene name in the “ECOLI” organism, and any associated EC numbers were downloaded. In the vast majority of cases, there was a one-to-one mapping from EC number to gene. However, some genes were associated with multiple EC number classifications (for example, multifunctional enzymes, and some EC numbers were coded for by multiple genes).

To ensure that our results were not biased by false positives (that is, nodes with an EC number that is linked to multiple genes where some were essential and others were not), we took a cautious approach and only considered nodes as essential if the associated EC number was solely coded for by an essential gene (*50*). Any nodes with an EC number that could be linked to multiple genes in the entire *E. coli* genome were classified as nonessential. We found that most EC numbers were unique to a single gene, with only 13 nodes linked to multiple essential and nonessential genes. This resulted in 61 of the 560 nodes being classified as essential in the metabolic network (table S5). It should be noted that the analysis performed in this work was also carried out for an alternative classification that required only a single linked gene to be essential for the node in the metabolic network to also be classified as essential. This led to similar results as presented here.

### Computational tools

Calculation of motif clustering coefficients, analysis of motif clustering type distributions, and extraction of subgraphs containing motif clusters were performed using mctools version 1.0. This software is open-source and freely available at https://github.com/BiocomputeLab/mctools.

## Supplementary Material

http://advances.sciencemag.org/cgi/content/full/4/3/eaap9751/DC1

## SUPPLEMENTARY MATERIALS

Supplementary material for this article is available at http://advances.sciencemag.org/cgi/content/full/4/3/eaap9751/DC1 text S1. Network data sets text S2. Motif clustering example text S3. Analysis of random network models fig. S1. FFL motifs extracted from the *A. fulgidus* metabolic network. fig. S2. FFL motifs extracted from the *E. coli* metabolic network. fig. S3. Expanded region of the *A. fulgidus* metabolic FFL motif cluster. fig. S4. FFL and FBL motif clustering types across many networks of metabolism. fig. S5. FFL motifs extracted from the transcriptional regulatory networks. fig. S6. FFL motifs extracted from the Little Rock Lake food web. fig. S7. FFL motifs extracted from the *C. elegans* neural network. fig. S8. FFL motifs extracted from the Wikipedia vote network. fig. S9. FFL motifs extracted from the air traffic control network. fig. S10. FFL motifs extracted from the Gnutella file-sharing network. fig. S11. FFL motifs extracted from the EU email network. fig. S12. Robustness of FFL clustering distributions for a selection of real-world networks to varying amounts of random edge removal. fig. S13. FFL motif clustering distributions for the Erdős-Rényi model. fig. S14. FFL motif clustering type distributions for the Erdős-Rényi model. fig. S15. Motif clustering type distributions for the node duplication model. table S1. General network statistics for the real-world systems. table S2. Motif-related statistics for FFLs in the real-world networks. table S3. Statistics comparing the original and extracted FFLs for the real-world systems. table S4. Results for motif clustering in random network models. table S5. Structural analysis of duplicated *E. coli* operon candidates. table S6. Essential EC numbers for the *E. coli* metabolic network.
