# Supplementary material for: Organization of feed-forward loop motifs reveals architectural principles in natural and engineered networks
Source: Sci Adv. 2018 Mar 28;4(3):eaap9751. doi: 10.1126/sciadv.aap9751 (PMC5903899; doi:10.1126/sciadv.aap9751)
Supplement: http://advances.sciencemag.org/cgi/content/full/4/3/eaap9751/DC1 [file supp_4_3_eaap9751__index.html]

Science Advances | Science Advances

## Supplementary Materials

**This PDF file includes:**

- text S1. Network data sets
- text S2. Motif clustering example
- text S3. Analysis of random network models
- fig. S1. FFL motifs extracted from the *A. fulgidus* metabolic network.
- fig. S2. FFL motifs extracted from the *E. coli* metabolic network.
- fig. S3. Expanded region of the *A. fulgidus* metabolic FFL motif cluster.
- fig. S4. FFL and FBL motif clustering types across many networks of metabolism.
- fig. S5. FFL motifs extracted from the transcriptional regulatory networks.
- fig. S6. FFL motifs extracted from the Little Rock Lake food web.
- fig. S7. FFL motifs extracted from the *C. elegans* neural network.
- fig. S8. FFL motifs extracted from the Wikipedia vote network.
- fig. S9. FFL motifs extracted from the air traffic control network.
- fig. S10. FFL motifs extracted from the Gnutella file-sharing network.
- fig. S11. FFL motifs extracted from the EU email network.
- fig. S12. Robustness of FFL clustering distributions for a selection of real-world networks to varying amounts of random edge removal.
- fig. S13. FFL motif clustering distributions for the Erdős-Rényi model.
- fig. S14. FFL motif clustering type distributions for the Erdős-Rényi model.
- fig. S15. Motif clustering type distributions for the node duplication model.
- table S1. General network statistics for the real-world systems.
- table S2. Motif-related statistics for FFLs in the real-world networks.
- table S3. Statistics comparing the original and extracted FFLs for the real-world systems.
- table S4. Results for motif clustering in random network models.
- table S5. Structural analysis of duplicated *E. coli* operon candidates.
- table S6. Essential EC numbers for the *E. coli* metabolic network.

Download PDF

**Files in this Data Supplement:**

- Adobe PDF - aap9751\_SM.pdf
