# Supplementary material for: Organization of feed-forward loop motifs reveals architectural principles in natural and engineered networks
Source: Sci Adv. 2018 Mar 28;4(3):eaap9751. doi: 10.1126/sciadv.aap9751 (PMC5903899; doi:10.1126/sciadv.aap9751)
Supplement: http://advances.sciencemag.org/cgi/content/full/4/3/eaap9751/DC1 [file aap9751_SM.pdf]

Science Advances

AAAS

advances.sciencemag.org/cgi/content/full/4/3/eaap9751/DC1

Supplementary Materials for

Organization of feed-forward loop motifs reveals architectural principles in natural and engineered networks

Thomas E. Gorochowski, Claire S. Grierson, Mario di Bernardo

Published 28 March 2018, *Sci. Adv.* **4**, eaap9751 (2018)
DOI: 10.1126/sciadv.aap9751

This PDF file includes:

- text S1. Network data sets
- text S2. Motif clustering example
- text S3. Analysis of random network models
- fig. S1. FFL motifs extracted from the *A. fulgidus* metabolic network.
- fig. S2. FFL motifs extracted from the *E. coli* metabolic network.
- fig. S3. Expanded region of the *A. fulgidus* metabolic FFL motif cluster.
- fig. S4. FFL and FBL motif clustering types across many networks of metabolism.
- fig. S5. FFL motifs extracted from the transcriptional regulatory networks.
- fig. S6. FFL motifs extracted from the Little Rock Lake food web.
- fig. S7. FFL motifs extracted from the *C. elegans* neural network.
- fig. S8. FFL motifs extracted from the Wikipedia vote network.
- fig. S9. FFL motifs extracted from the air traffic control network.
- fig. S10. FFL motifs extracted from the Gnutella file-sharing network.
- fig. S11. FFL motifs extracted from the EU email network.
- fig. S12. Robustness of FFL clustering distributions for a selection of real-world networks to varying amounts of random edge removal.
- fig. S13. FFL motif clustering distributions for the Erdős-Rényi model.
- fig. S14. FFL motif clustering type distributions for the Erdős-Rényi model.
- fig. S15. Motif clustering type distributions for the node duplication model.
- table S1. General network statistics for the real-world systems.
- table S2. Motif-related statistics for FFLs in the real-world networks.
- table S3. Statistics comparing the original and extracted FFLs for the real-world systems.

- table S4. Results for motif clustering in random network models.
- table S5. Structural analysis of duplicated *E. coli* operon candidates.
- table S6. Essential EC numbers for the *E. coli* metabolic network.

S1 Network data sets

We considered a wide range of directed networks generated from biological, technological and social systems. General network statistics for each of these and are presented in table S1.

- *E. coli Transcription* – Network of transcriptional regulation in *E. coli* [4]. Nodes represent operons and an edge exists from node i to j if any product of operon i regulates operon j in some way. We ignore the specific type of regulation (e.g., positive or negative).
- *S. cerevisiae Transcription* – Network of transcriptional regulation in *S. cerevisiae* based on the YPD database [2]. Interactions between transcription factor proteins and genes are included. Nodes represent transcription factors and genes, with an edge from node i to j if i regulates j. Each protein complex of transcription factors is represented by a single node and we ignore the specific type of regulation (e.g., positive or negative).
- *E. coli and A. fulgidus Metabolism* – Networks of interactions between metabolic enzymes in *E. coli* and *A. fulgidus* [24]. Nodes represent metabolic enzymes and an edge exists from node i to j if enzyme i catalyses a reaction whose product is used as a substrate for enzyme j.
- *C. elegans Neural* – Neural network of *C. elegans* [25]. Nodes represent neurons and edges directed connections (synapses) between neurons.
- *Gnutella File Sharing* – Snapshot of the Gnutella peer-to-peer file sharing network from 4th August 2002 [20]. Nodes represent hosts in the Gnutella network and edges directed connections between hosts.
- *Air Traffic Control* – This network was taken from `http://research.mssm.edu/maayan/datasets/qualitative_networks.shtml` and constructed using the FAA (Federal Aviation Administration) National Flight Data Centre (NFDC), Preferred Routes Database (`http://www.fly.faa.gov`). Nodes represent airports or service centres and directed edges are created from strings of preferred routes recommended by the NFDC.
- *Little Rock Lake Food Web* – Food web of the Little Rock lake in Wisconsin, first published by Martinez [23]. Nodes represent autotrophs, herbivores, carnivores or decomposers and edges predation/food sources. An edge exists from node i to j if i is eaten by j.
- *EU E-mail* – Network generated from email logs of a large European research institution between October 2003 to May 2005 [22]. Nodes represent e-mail addresses and an edge exists from node i to j if at least one message has been sent from i to j.
- *Wikipedia Vote* – Network containing all users and discussions from the inception of Wikipedia until January 2008 [21]. Nodes represent users and an edge exists from node i to j if user i voted on user j.

S2 Motif clustering example

In this section we aim to help clarify the individual steps required to calculate each form of motif clustering (general, homologous and heterologous) for a simple network. Our example network of interest $\mathcal{G} = (\mathcal{V}, \mathcal{E})$ is defined as,

$$\begin{aligned}\mathcal{V} &= \{v_1, v_2, v_3, v_4, v_5, v_6, v_7, v_8\}\\ \mathcal{E} &= \{(v_1, v_2), (v_1, v_3), (v_1, v_4), (v_2, v_4), (v_2, v_5), (v_3, v_4), (v_4, v_7), (v_5, v_8),\\ &\quad (v_6, v_4), (v_7, v_5), (v_7, v_6), (v_8, v_7)\}\end{aligned}$$

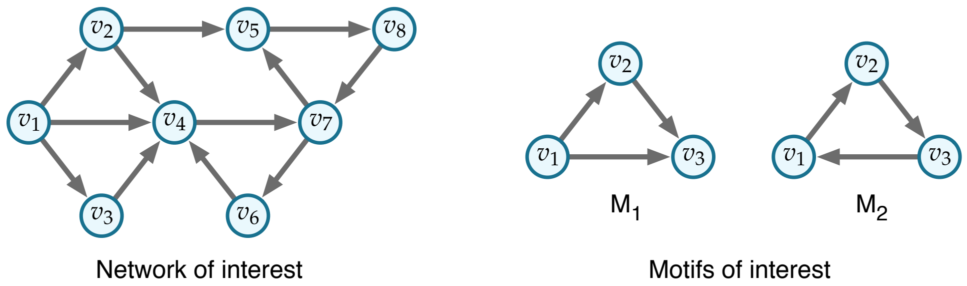

To cover all possible types of motif clustering we consider the motif clustering of both the feed-forward loop (FFL) and feedback loop (FBL). Our set of motif types is therefore given by

$$\mathcal{M} = \{M_1, M_2\},$$

where,

$$\begin{aligned}M_1 = (\mathcal{V}_1^M, \mathcal{E}_1^M), \quad \mathcal{V}_1^M = \{v_1, v_2, v_3\}, \quad \mathcal{E}_1^M = \{(v_1, v_2), (v_1, v_3), (v_2, v_3)\}\\ M_2 = (\mathcal{V}_2^M, \mathcal{E}_2^M), \quad \mathcal{V}_2^M = \{v_1, v_2, v_3\}, \quad \mathcal{E}_2^M = \{(v_1, v_2), (v_2, v_3), (v_3, v_1)\}\end{aligned}$$

Searching through the network $\mathcal{G}$ for sub-isomorphisms of each of the motifs in $\mathcal{M}$, we find

$$F = \{\underbrace{\{v_1, v_2, v_4\}}_{f_1}, \underbrace{\{v_1, v_3, v_4\}}_{f_2}, \underbrace{\{v_4, v_7, v_6\}}_{f_3}, \underbrace{\{v_7, v_5, v_8\}}_{f_4}\}$$

The general motif clustering is then given by,

$$\begin{aligned}M_c &= \frac{|f_1 \cap f_2| + |f_1 \cap f_3| + |f_1 \cap f_4| + |f_2 \cap f_3| + |f_2 \cap f_4| + |f_3 \cap f_4|}{2+2+2+2+2+2}\\ &= \frac{2+1+0+1+0+1}{12}\\ &= \frac{5}{12}\end{aligned}$$

For homologous and heterologous motif clustering we are required to partition the set of found motifs into sets corresponding to each of the motifs in $\mathcal{M}$. Therefore,

$$F = F_1 \cup F_2$$

where F_1 relates to nodes taking part in FFLs in $\mathcal{G}$, and F_2 relates to nodes taking part in FBLs in $\mathcal{G}$, giving,

$$F_1 = \{\underbrace{\{v_1, v_2, v_4\}}_{f_{11}}, \underbrace{\{v_1, v_3, v_4\}}_{f_{12}}\}, \qquad F_2 = \{\underbrace{\{v_4, v_7, v_6\}}_{f_{21}}, \underbrace{\{v_7, v_5, v_8\}}_{f_{22}}\}.$$

Using these sets it is then possible to calculate the homologous motif clustering as,

$$\begin{aligned} M_c^{+} &= \frac{|f_{11} \cap f_{12}| + |f_{21} \cap f_{22}|}{2+2} \\ &= \frac{2+1}{4} \\ &= \frac{3}{4} \end{aligned}$$

and heterologous motif clustering as,

$$\begin{aligned} M_c^{\pm} &= \frac{|f_{11} \cap f_{21}| + |f_{11} \cap f_{22}| + |f_{12} \cap f_{21}| + |f_{12} \cap f_{22}|}{2+2+2+2} \\ &= \frac{1+0+1+0}{8} \\ &= \frac{2}{8} \end{aligned}$$

S3 Analysis of random network models

To better understand motif clustering and motif clustering type distributions in some idealised situations, we analyzed their behaviour for two random network models. Specifically, a standard Erdős-Rényi model to give an unbiased view and a node duplication model to capture a possible generative mechanism.

S3.1 Erdős-Rényi model

We first considered a standard directed Erdős-Rényi model where every directed edge in the network has an independent fixed probability of p_e. Although this is unlikely to be an accurate model for many real-world systems, it permits mathematical analysis and was useful as a foundation on which to make comparisons between different types of motif clustering without bias.

To study the effect that the size of the network n (nodes) and edge probability p_e had on motif clustering, we computationally generated a large sample of random networks with varying parameters. In particular, we sampled 1000 networks that contained a fixed size of 1000 nodes with various edge probabilities p_e = 0.005 $\approx$ 5000 directed edges, 0.01 $\approx$ 10000 directed edges, and 0.02 $\approx$ 20000 directed edges, and also sampled 1000 networks with a fixed edge probability p_e = 0.005 and various network sizes, n = 1000, 1500 and 2000 nodes. While the focus here is on the FFL motif due to its significant expression in our networks of interest. It should be noted that the analysis we carry out can be easily be extended to alternative types of motif.

For each of the randomly generated networks, we calculated the motif clustering coefficient for the FFL motif (fig. S13). We found that as network size and edge probability increased, the motif clustering coefficient decreased. For a fixed edge probability and increasing network size (fig. S13B), this can be explained by the number of edges (related to the number of FFLs) growing at a rate n, while the total possible number of edges increases like n^2. This discrepancy leads to a decreasing density of FFLs and therefore a lower motif clustering coefficient as the probability of sharing a node becomes reduced.

For a fixed network size and increasing edge probability (fig. S13A), the reduced motif clustering can be explained in a similar way. As the edge probability increases so too does the number of FFLs[1]. This larger density increases the expected number of pairs of FFL that share nodes. However, as the number of FFLs m increases, the total possible number of pairs of FFL also increases, but at a faster rate of m^2. As before, this difference leads to an overall reduction in the motif clustering coefficient.

We also analysed the motif clustering type distributions for the FFL motif (fig. S14). This showed a dependency between the number of FFLs sharing one node, $\mathbb{M}_1$ (types 1 to 6), and two nodes, $\mathbb{M}_2$ (types 7 to 12), on the size of the network and the edge probability. Specifically, the ratio of types sharing one node to two nodes defined as $\mathbb{M}_1/\mathbb{M}_2$, increased with network size and edge probability. Furthermore, variation was seen between types even though they shared the same number of nodes. For example, types 1, 4 and 6 were half the fraction of types 2, 3 and 5, and types

[1] An increasing edge probability p_e will only lead to increasing numbers of FFL motifs up to some threshold, in this case for $p_e \leq$ 0.5. Generally, most complex networks are sparse with small p_e, therefore this assumption will hold.

7, 10 and 12 were half the fraction of types 8, 9 and 11.

To better understand these relationships between motif clusters with one and two node overlaps, we calculated the number of pairs of motif clustered sharing one $\mathbb{M}_1$ and two $\mathbb{M}_2$ nodes in terms of the network size n and the probability of a directional edge p_e. Considering any three node motif this gives,

$$\mathbb{M}_1(n, p_e) = \binom{n}{5} P_1(p_e) \tag{S1}$$

$$\mathbb{M}_2(n, p_e) = \binom{n}{4} P_2(p_e) \tag{S2}$$

where $P_1(p_e)$ is the probability of five randomly chosen nodes leading to a motif cluster type sharing one node, and $P_2(p_e)$ is the probability of four randomly chosen nodes leading to a motif clustering type sharing two nodes. Using these we derive the following scaling relationship,

$$\frac{\mathbb{M}_1(n, p_e)}{\mathbb{M}_2(n, p_e)} = \frac{P_1(p_e)(n-4)}{5P_2(p_e)} \tag{S3}$$

$$\sim \frac{P_1(p_e)}{P_2(p_e)} \times n \tag{S4}$$

This highlights that for an Erdős-Rényi model, motif clusters sharing a single node will eventually dominate those sharing two as the network size n increases. Exactly when this happens depends on the relative probabilities of motif clusters sharing one or two nodes, P_1 and P_2, and the probability of an edge p_e.

Using this relationship we can now apply it to the FFL and attempt to reconcile the inter-type difference seen in fig. S14. We calculated $\mathbb{M}_1^{FLL}$ and $\mathbb{M}_2^{FLL}$ by enumerating all possible clustering types (Fig. 2A in the main text), and then derived expressions for the probability of finding five randomly selected nodes producing a FFL motif cluster sharing a single node, P_1^{FFL}, and finding four randomly selected nodes producing a FFL motif cluster sharing two nodes, P_2^{FFL}. These are given by,

$$P_1^{FFL}(p_e) = \left[\underbrace{6\binom{5}{3}}_{t_1} + \underbrace{12\binom{5}{3}}_{t_2} + \underbrace{12\binom{5}{3}}_{t_3} + \underbrace{6\binom{5}{3}}_{t_4} + \underbrace{12\binom{5}{3}}_{t_5} + \underbrace{6\binom{5}{3}}_{t_6} \right] p_1 \tag{S5}$$

$$P_2^{FFL}(p_e) = \left[\underbrace{2\binom{4}{2}}_{t_7} + \underbrace{6 \times 4}_{t_8} + \underbrace{6 \times 4}_{t_9} + \underbrace{2\binom{4}{2}}_{t_{10}} + \underbrace{6 \times 4}_{t_{11}} + \underbrace{2\binom{4}{2}}_{t_{12}} \right] p_2 \tag{S6}$$

where, $p_5 = [p_e(1-p_e)]^5$ is the probability of picking five unidirectional edges between four nodes, $p_6 = [p_e(1-p_e)]^6$ is the probability of picking six unidirectional edges between five nodes, and the contributions from each FFL motif cluster type t_i for $i = 1 \ldots 12$ are separately highlighted for clarity. These individual type contributions relate to the number of ways that a particular type of motif can arise and accounts for differences in the specific numbers of types.

Substituting Eqs. (S5) and (S6) into (S3), we find the following relationship for FFLs

$$\frac{\mathbb{M}_1^{FFL}(n, p_e)}{\mathbb{M}_2^{FFL}(n, p_e)} = (4 - n)(p_e - 1)p_e \tag{S7}$$

As expected this relationship is in close agreement with results calculated using the computationally generated samples shown in Table S4.

S3.2 Node duplication model

The second random network model we considered was based on random node duplication from a template motif. Starting with a given motif, we randomly picked a single node (with all nodes having equal probability), and duplicated it including any inward and outward edges. This process continued until a specified number of duplications had taken place. The only variable in this model is the final size of the network generated. This type of network model is relevant to motif clustering because duplication of a single node (with connections) is a way of generating a pair of fully clustered motifs. Any motifs that the original node took part in are duplicated for the new node and also share the maximum possible number of nodes (i.e., they are fully clustered). Furthermore, it is known that duplication and divergence is a common process by which many natural systems evolve, and so it was important to investigate how this process might affect motif clustering features.

As with the Erdős-Rényi model, we focused on the feed-forward loop motif. We carried out 27, 37 and 47 random duplication steps to give resultant networks of 30, 40 and 50 nodes. Furthermore, 1000 networks were independently generated for each case to better understand the variance in these results.

Figure S15A shows the distribution of motif clustering coefficients as the size of the network grows. As with the Erdős-Rényi model we also see a decrease in motif clustering with network size. This can be accounted for by each newly duplicated node generating a far greater numbers of pairs of motif that share one node versus two. As the size of the network increases this is compounded and the overall motif clustering decreases.

Resultant motif clustering type distributions of these networks are shown in fig. S15B. Interestingly, these display a complementary distribution to the Erdős-Rényi model, with only motif clustering types 1, 4, 6, 7, 10 and 12 being present. In the Erdős-Rényi model these types saw under-expression in comparison to similar clustering types that share the same number of nodes. This feature can be attributed to growth of the network occurring through duplication alone. This process ensures that duplicated nodes maintain the same role as the parent. In the case of a FFL motif each node can be classified as either an input, intermediate or output. Duplication of a node maintains these types and so only those motif clustering types where each node has a unique role are possible. Motif clustering types 2, 3, 5, 8, 9 and 11 that are not found contain nodes where a role is mixed between the two single FFLs, for example acting as an input in one FFL and an intermediate node in the other.

As with the Erdős-Rényi model we also see a discrepancy in the quantities of motif clustering types that have one, $\mathbb{M}_1$ (types 1 to 6), and two, $\mathbb{M}_2$ (types 7 to 12), node overlaps. Table S4 shows

that the ratio of motif clustering types with one and two shared nodes, $\mathbb{M}_1/\mathbb{M}_2$, increases linearly, scaling at a rate of $\frac{1}{3}$.

To analyse the precise structure of this distribution we calculated the expected number of FFLs in relation to the number of nodes, n. With every FFL comprising of three types of node: input, intermediate and output, and because each node type has equal probability of duplication, we would expect $\frac{n}{3}$ nodes of each type to occur. Furthermore, because every node of a particular type (e.g., input), must be connected to every other node of a different type (e.g., intermediate and output), we would expect the total number of FFLs to be given by,

$$\frac{n}{3} \times \frac{n}{3} \times \frac{n}{3} = \frac{n^3}{27} \tag{S8}$$

The total number of possible clustered motifs is the number of unique pairs of motif that can be selected from this set. Specifically,

$$\binom{n^3/27}{2} \tag{S9}$$

where $\binom{n}{2}$ is a binomial coefficient calculating the number of ways of picking two elements from a set of size n without taking order into account. Because the FFL contains three nodes, this set can be partitioned into the numbers of motifs that have node overlaps of none ($\mathbb{M}_0$), one ($\mathbb{M}_1$) and two ($\mathbb{M}_2$), and can therefore be written as,

$$\binom{n^3/27}{2} = \mathbb{M}_0(n) + \mathbb{M}_1(n) + \mathbb{M}_2(n) \tag{S10}$$

To calculate the number of pairs of motif that share exactly two nodes $\mathbb{M}_2$, we consider a particular input and intermediate node. These two nodes by definition are expected to connect to $\frac{n}{3}$ output nodes, each of which defines a unique FFL. Furthermore, because the input and intermediate node are shared between all these motifs they contribute $\binom{n/3}{2}$ possible clustered motifs with two shared nodes. By considering all possible input and intermediate nodes, of which there are $\frac{n}{3} \times \frac{n}{3} = \frac{n^2}{9}$, and the three ways that two node types can be chosen, we have,

$$\begin{aligned} \mathbb{M}_2(n) &= 3 \times \frac{n^2}{9} \times \binom{n/3}{2} \\ &= \frac{(n-3)n^3}{54} \end{aligned} \tag{S11}$$

To calculate the number of pairs of FFL that share exactly one node $\mathbb{M}_1$, we can use a similar approach, but only consider a single input node. In this case, there are $\frac{n}{3} \times \frac{n}{3} = \frac{n^2}{9}$ FFLs that share this node and so its contribution to the motif clustering is $\binom{n^2/9}{2}$. Sharing a single input node does not exclude the possibility of sharing two nodes and so these must be subtracted in addition to

considering the three types of node that can be chosen. This gives,

$$\begin{aligned}\mathbb{M}_1(n) &= 3 \times \frac{n}{3} \times \binom{n^2/9}{2} - 3 \times \frac{n^2}{9} \times \binom{n/3}{2} \\ &= \frac{(n-3)n^4}{162}.\end{aligned} \tag{S12}$$

Finally, the number of clustered motifs with no shared nodes $\mathbb{M}_0$ can be calculated through substitution of Eqs. (S11) and (S12) into (S10),

$$\begin{aligned}\mathbb{M}_0(n) &= \binom{n^3/27}{2} - 3 \times \frac{n}{3} \times \binom{n^2/9}{2} \\ &= \frac{(n^3 - 9n^2 + 54)n^3}{1458}\end{aligned} \tag{S13}$$

We are now in a position to better understand how the proportion of motif clustering types sharing one and two nodes varies with the size of the network. Specifically, we find the following scaling law:

$$\frac{\mathbb{M}_1(n)}{\mathbb{M}_2(n)} \propto \frac{n}{3} \tag{S14}$$

This illustrates that as the size of the network n grows, the proportion of clustering types with two shared nodes (types 7, 10 and 12), will rapidly decrease in comparison to those types only sharing a single node (types 1, 4 and 6). This analysis is also in agreement with the rate of $\frac{1}{3}$ estimated previously from computational samples of random networks using this model (Table S4).

While here we have analysed the FFL a similar approach can be used for all possible starting motifs. In these cases, biases may arise due to the inherent symmetries of a given motif causing multiple nodes to have the same role.

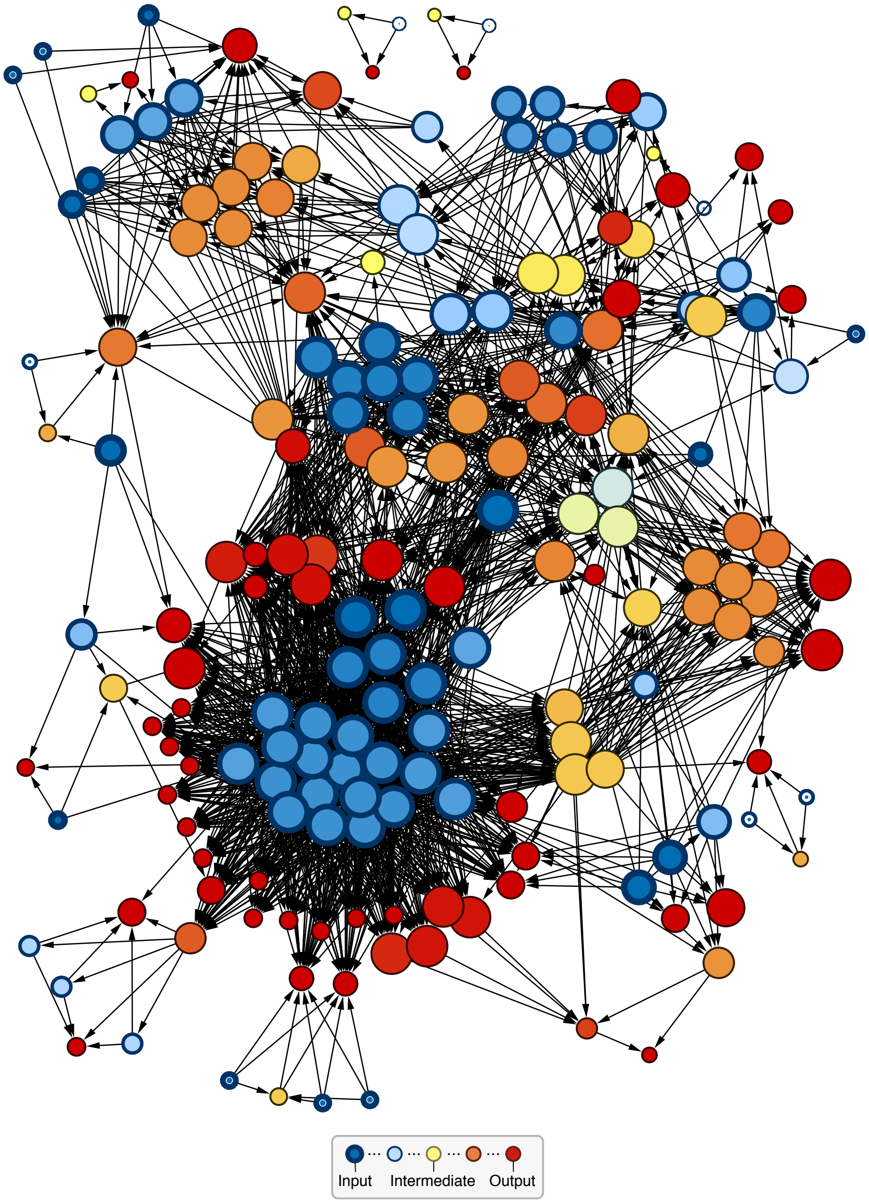

fig. S1. Feed-forward motifs extracted from the *A. fulgidus* metabolic network. Node size corresponds to motif clustering diversity (MCD) and colour represents the node spin. Inputs with $S = -1$ are blue (with thick edge), intermediate nodes with $S = 0$ are yellow, and output nodes with $S = 1$ are red.

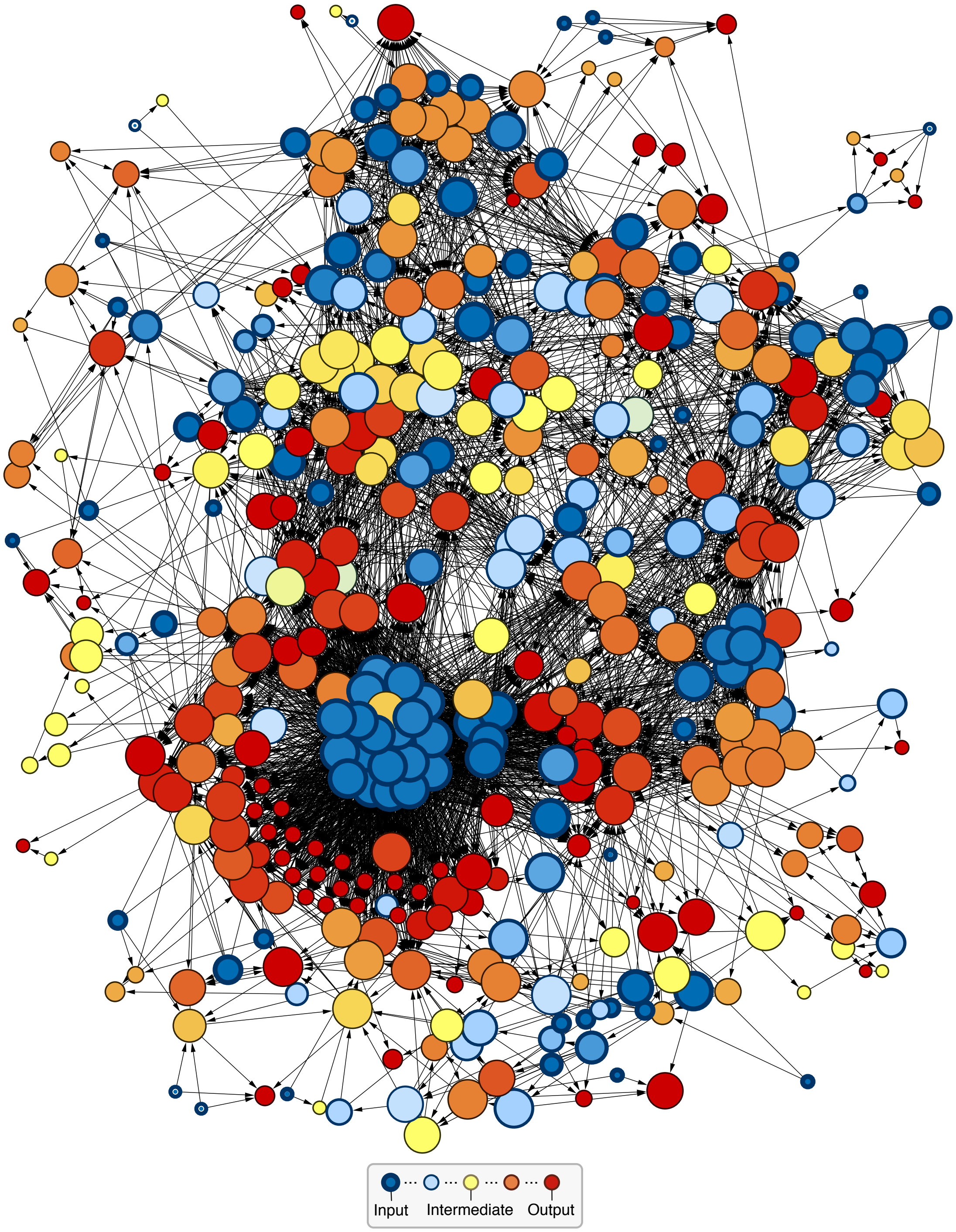

fig. S2. Feed-forward motifs extracted from the *E. coli* metabolic network. Node size corresponds to motif clustering diversity (MCD) and colour represents the node spin. Inputs with $S = -1$ are blue (with thick edge), intermediate nodes with $S = 0$ are yellow, and output nodes with $S = 1$ are red.

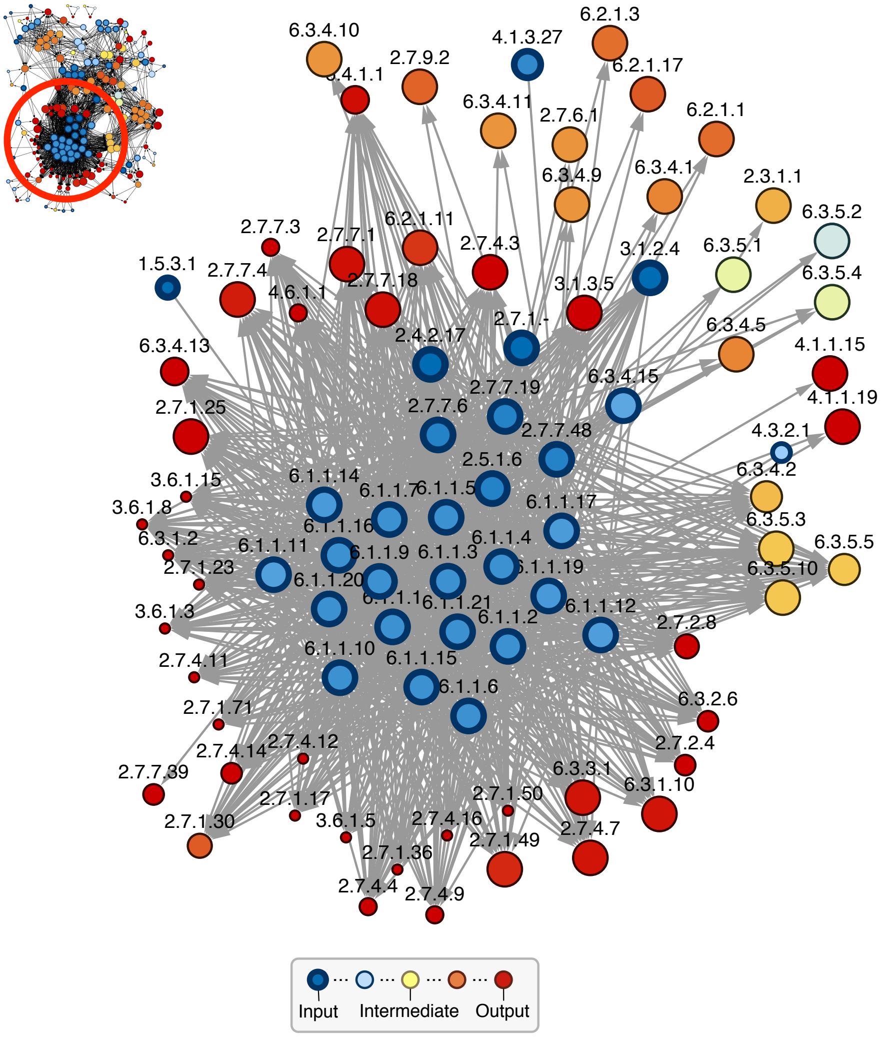

EC Number	Description	General Role
2.4.2.17	ATP phosphoribosyltransferase	RNA synthesis
2.5.1.6	Methionine adenosyltransferase	DNA/RNA/Protein synthesis
2.7.1.–	Phosphotransferases with an alcohol group as acceptor	Energy storage
2.7.7.6	DNA-directed RNA polymerase	RNA synthesis
2.7.7.19	Polynucleotide adenylyltransferase	RNA synthesis
2.7.7.48	RNA-directed RNA polymerase	RNA synthesis
3.1.2.4	3-hydroxyisobutyryl-CoA hydrolase	Protein recycling
6.1.1.–	Ligases Forming Aminoacyl-tRNA and Related Compounds	Protein synthesis
6.3.4.15	Biotin-(acetyl-CoA-carboxylase) ligase	Growth and protein recycling

fig. S3. Expanded region of the *A. fulgidus* metabolic feed-forward loop motif cluster. Individual node (enzyme) classifications have been included in the form of Enzyme Commission (EC) numbers. Network in top left highlights the region of the motif cluster that has been expanded. Table gives EC descriptions for the some of most highly connected nodes found in the central region of the cluster.

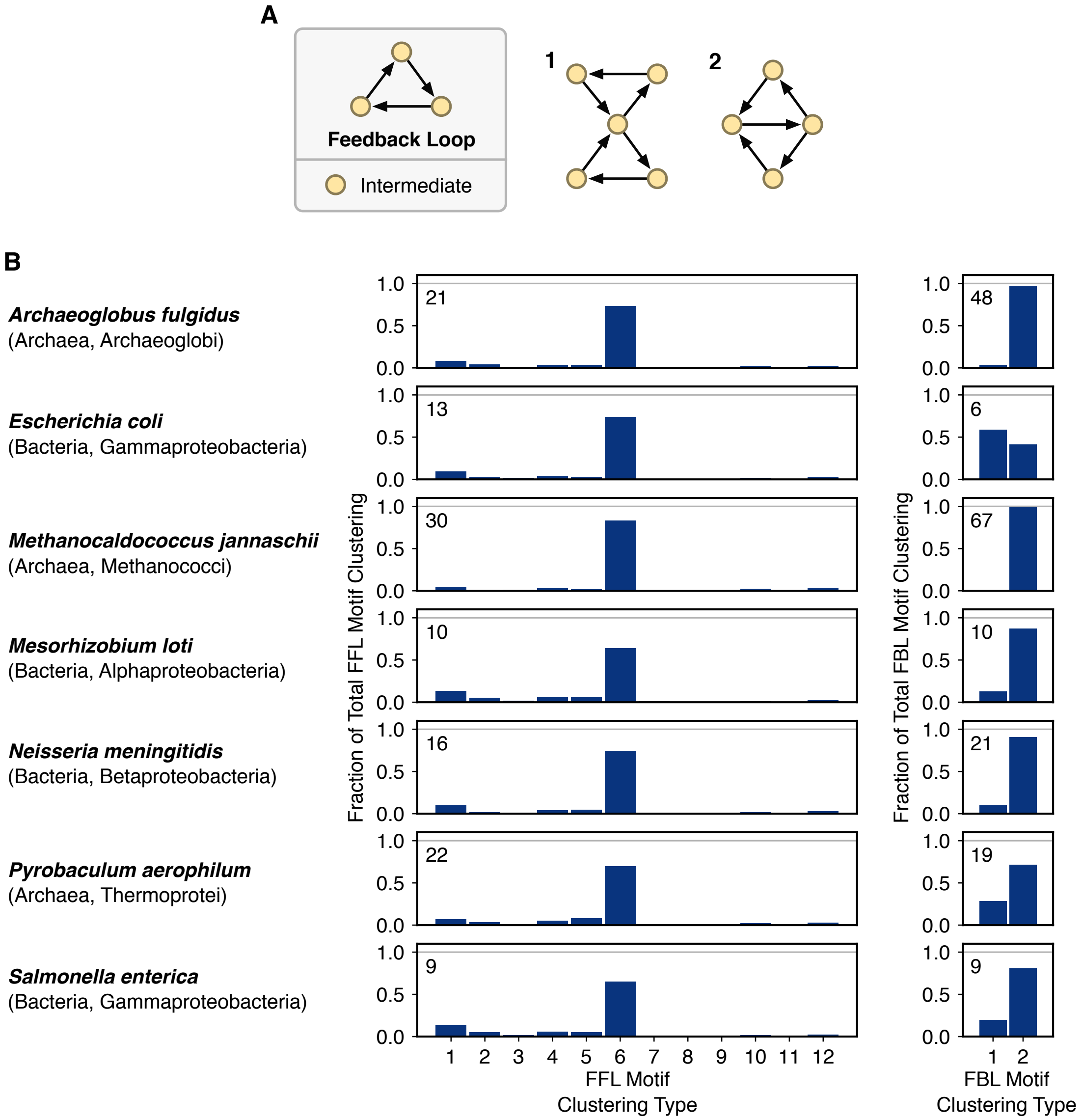

fig. S4. FFL and FBL motif clustering types across many networks of metabolism. (**A**) The two unique motif clustering types for two feedback loops (FBLs). Due to the balanced number of input and output edges for each node, every node acts as an intermediate. (**B**) Motif cluster distributions for FFLs (left) and FBLs (right) across seven diverse networks of metabolism. *A. fulgidus* and *E. coli* from the main study have also been included for comparison. Numbers in the top left corner of each plot show the percentage of FFLs or FBLs clustered in the network (i.e., not found completely isolated). Note the very low percentage of clustered motifs for the *E. coli* network (58/861 = 6%) suggesting FBL clustering plays a less important role.

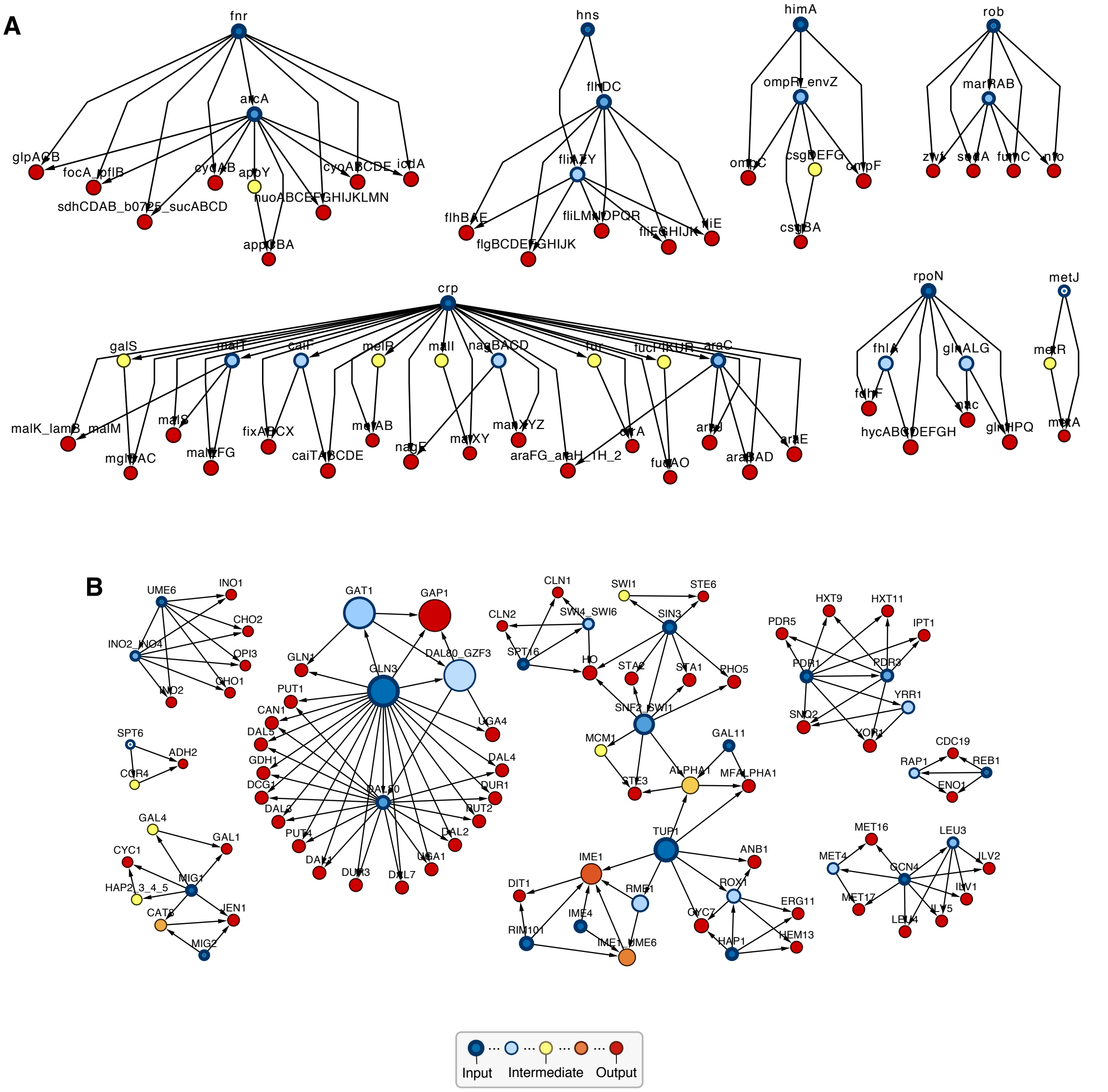

fig. S5. Feed-forward motifs extracted from the transcriptional regulatory networks. (**A**) *E. coli* transcriptional regulation network. (**B**) *S. cerevisiae* transcriptional regulation network. Node size corresponds to motif clustering diversity (MCD) and colour represents the node spin. Inputs with $S = -1$ are blue (with thick edge), intermediate nodes with $S = 0$ are yellow, and output nodes with $S = 1$ are red. Operon and gene names have also been included. A hierarchical layout was used to highlight this structure within the extracted FFLs.

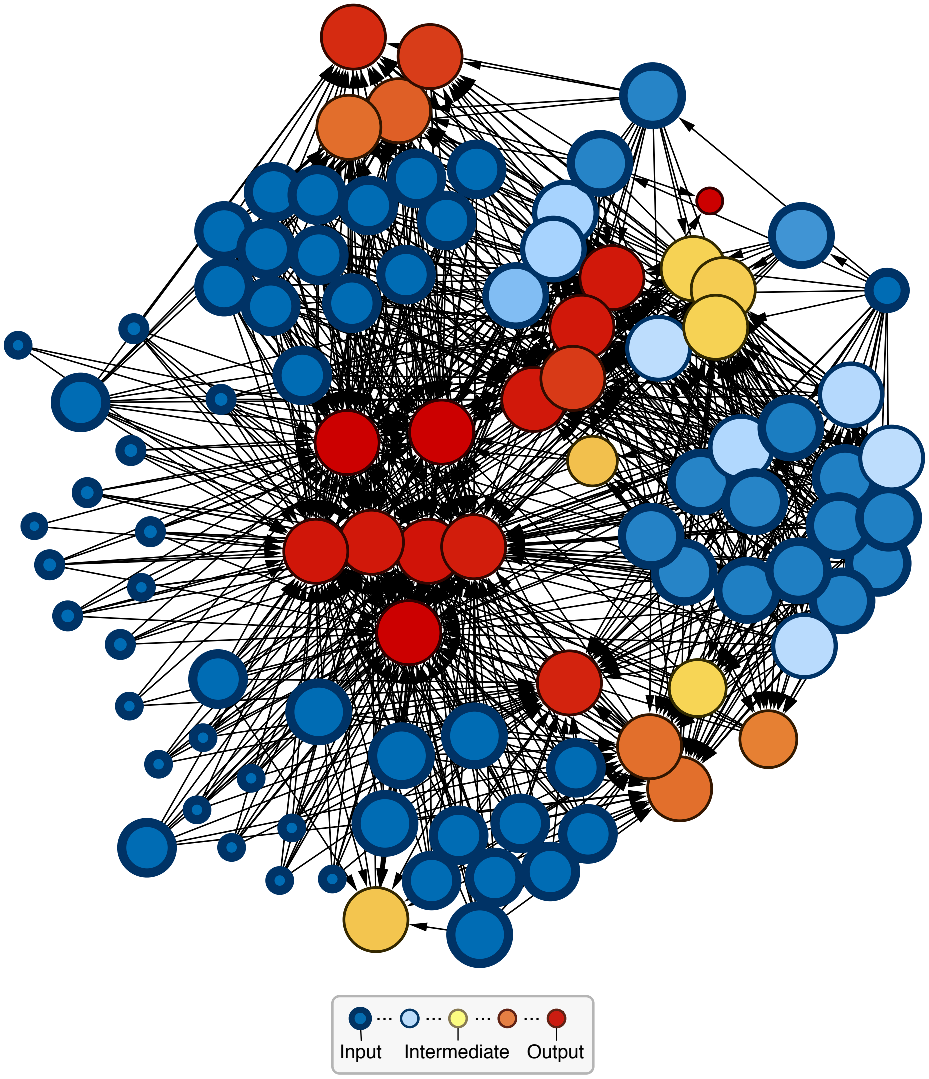

fig. S6. Feed-forward motifs extracted from the Little Rock Lake food web. Node size corresponds to motif clustering diversity (MCD) and colour represents the node spin. Inputs with $S = -1$ are blue (with thick edge), intermediate nodes with $S = 0$ are yellow, and output nodes with $S = 1$ are red.

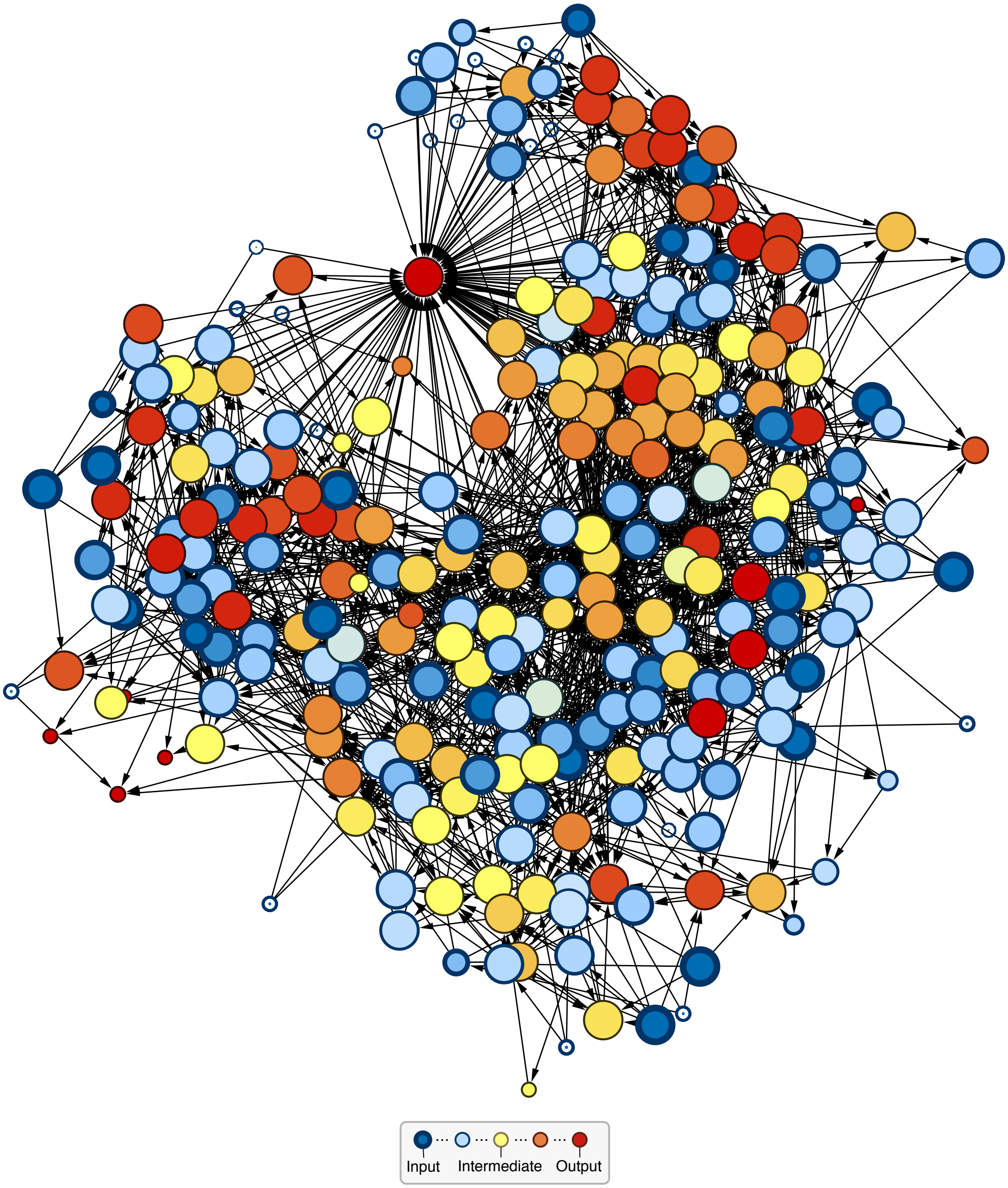

fig. S7 Feed-forward motifs extracted from the *C. elegans* neural network. Node size corresponds to motif clustering diversity (MCD) and colour represents the node spin. Inputs with $S = -1$ are blue (with thick edge), intermediate nodes with $S = 0$ are yellow, and output nodes with $S = 1$ are red.

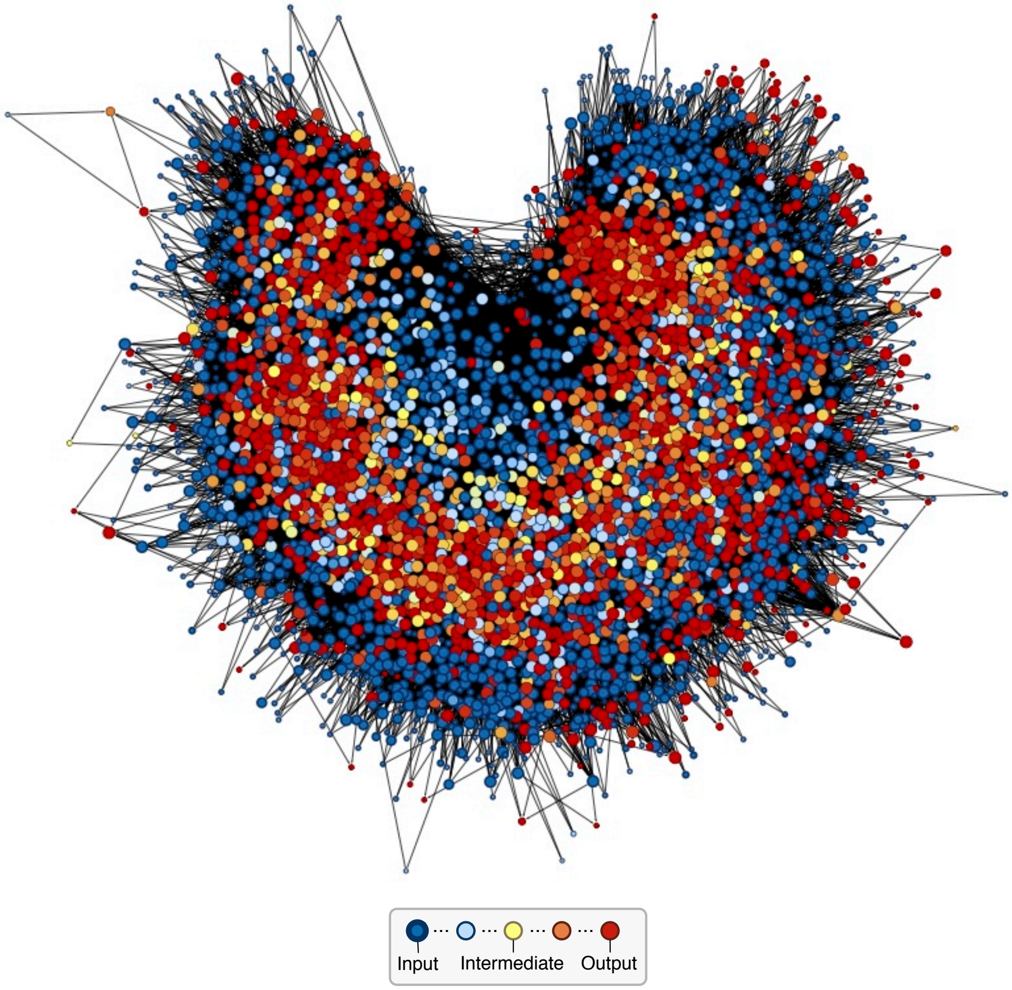

fig. S8. Feed-forward motifs extracted from the Wikipedia vote network. Node size corresponds to motif clustering diversity (MCD) and colour represents the node spin. Inputs with $S = -1$ are blue (with thick edge), intermediate nodes with $S = 0$ are yellow, and output nodes with $S = 1$ are red.

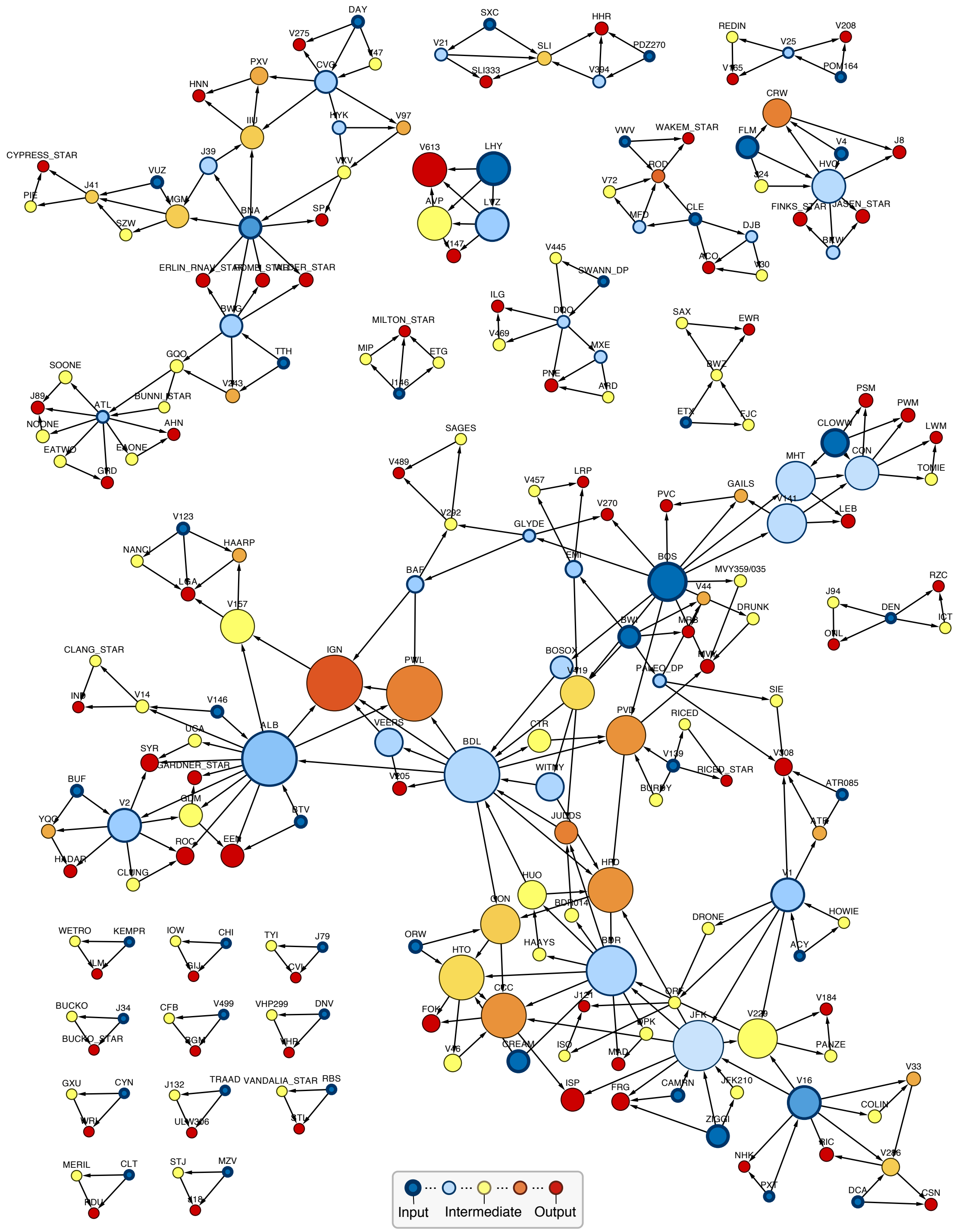

fig. S9. Feed-forward motifs extracted from the air traffic control network. Node size corresponds to motif clustering diversity (MCD) and colour represents the node spin. Inputs with $S = -1$ are blue (with thick edge), intermediate nodes with $S = 0$ are yellow, and output nodes with $S = 1$ are red.

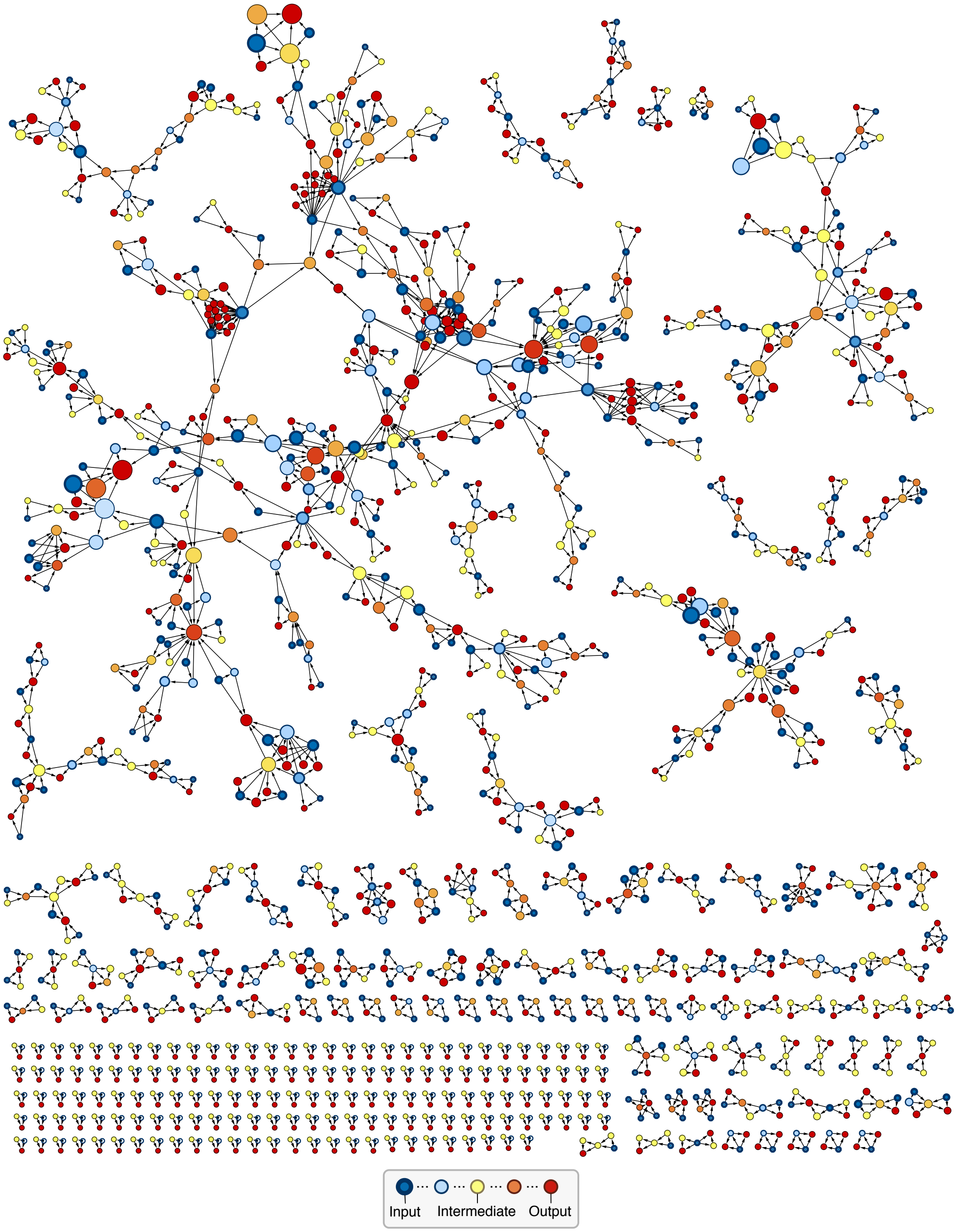

fig. S10. Feed-forward motifs extracted from the Gnutella file sharing network. Node size corresponds to motif clustering diversity (MCD) and colour represents the node spin. Inputs with $S = -1$ are blue (with thick edge), intermediate nodes with $S = 0$ are yellow, and output nodes with $S = 1$ are red.

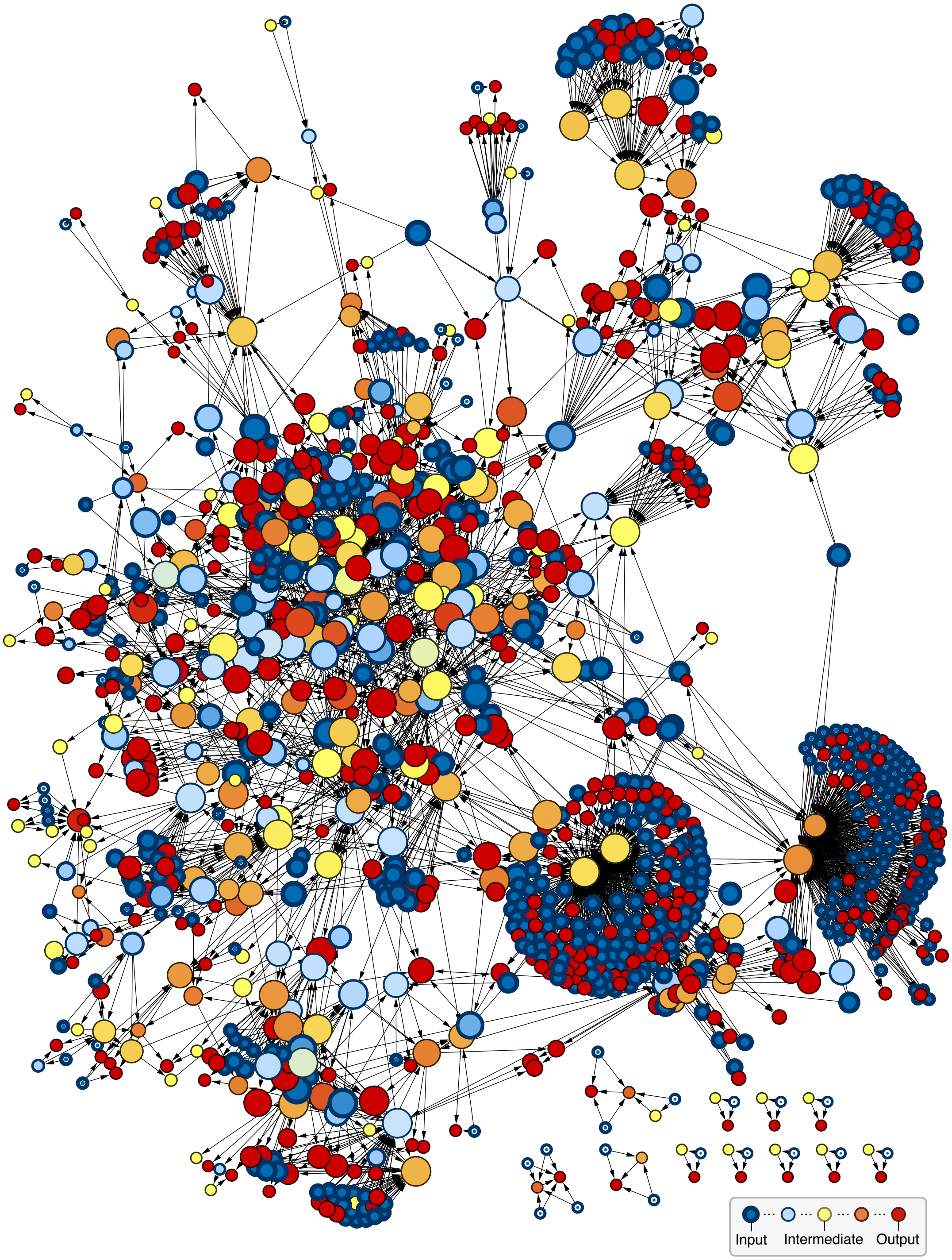

fig. S11.Feed-forward motifs extracted from the EU e-mail network. Node size corresponds to motif clustering diversity (MCD) and colour represents the node spin. Inputs with $S = -1$ are blue (with thick edge), intermediate nodes with $S = 0$ are yellow, and output nodes with $S = 1$ are red.

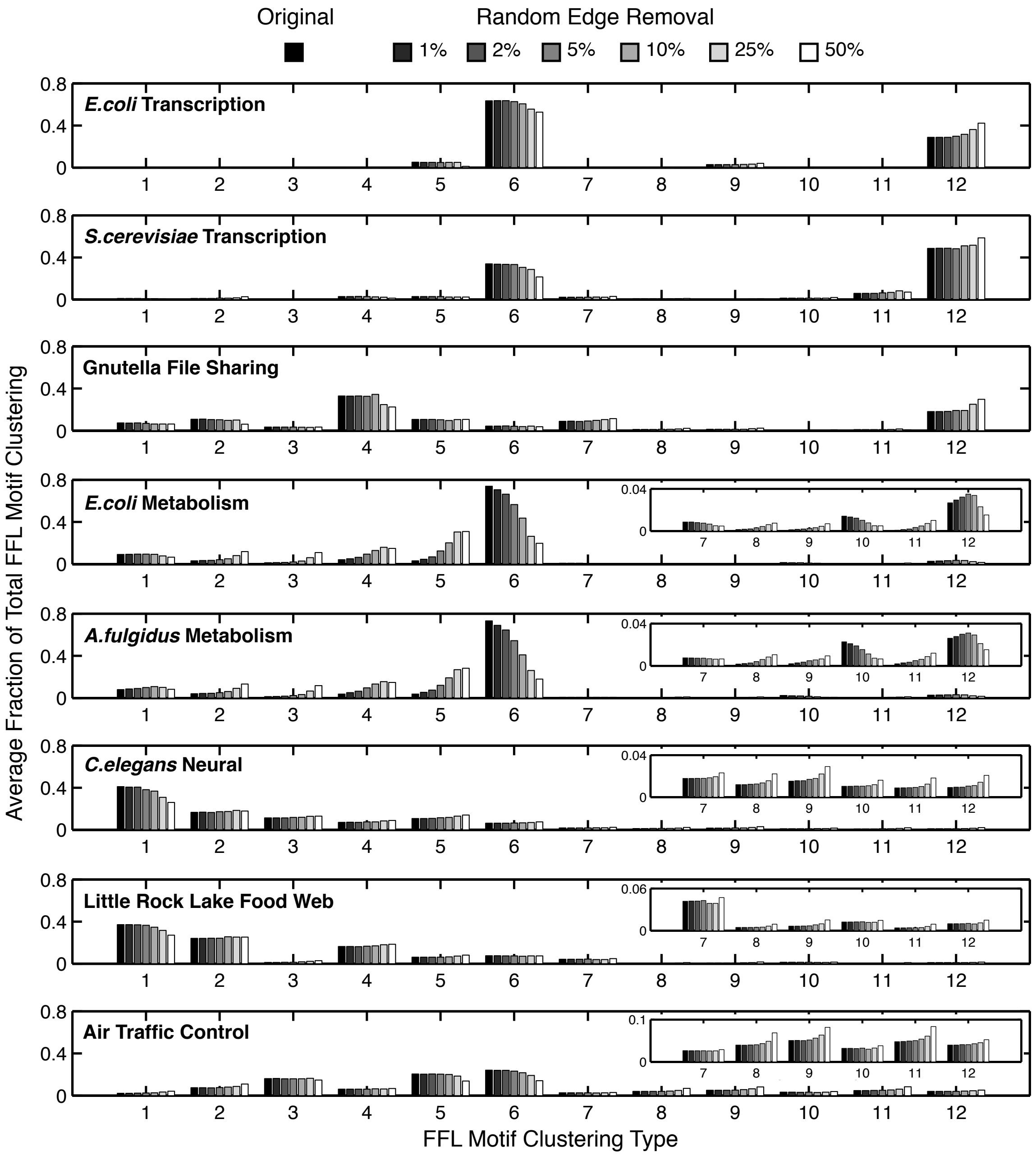

fig. S12. Robustness of feed-forward loop clustering distributions for a selection of real-world networks to varying amounts of random edge removal. For each edge removal percentage of 1, 2, 5, 10, 25 and 50% we generated 500 networks and calculated an average distribution. Inserts have been included for distributions where motif clustering types 7–12 display very low overall fractions in comparison to types 1–6.

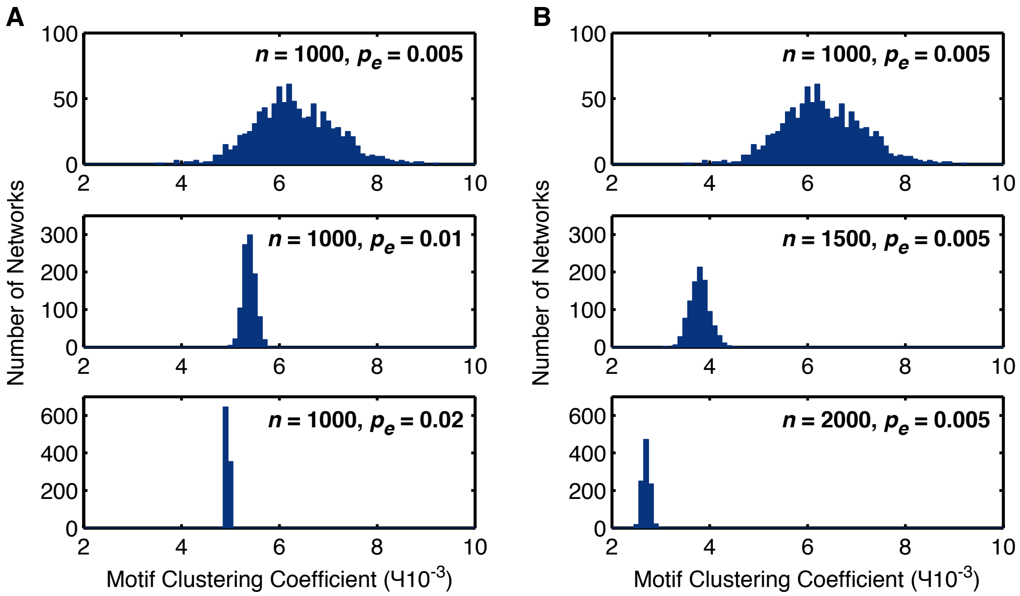

fig. S13. Feed-forward loop motif clustering distributions for the Erdos̋-Renýi model. We analysed the effect of varying both network size n and edge probability p_e, and calculated 1000 random networks for each scenario. (**A**) Fixed size network of 1000 nodes and several edge probabilities of 0.005, 0.01 and 0.02. (**B**) Fixed edge probability of 0.005 and several network sizes of 1000, 1500 and 2000 nodes.

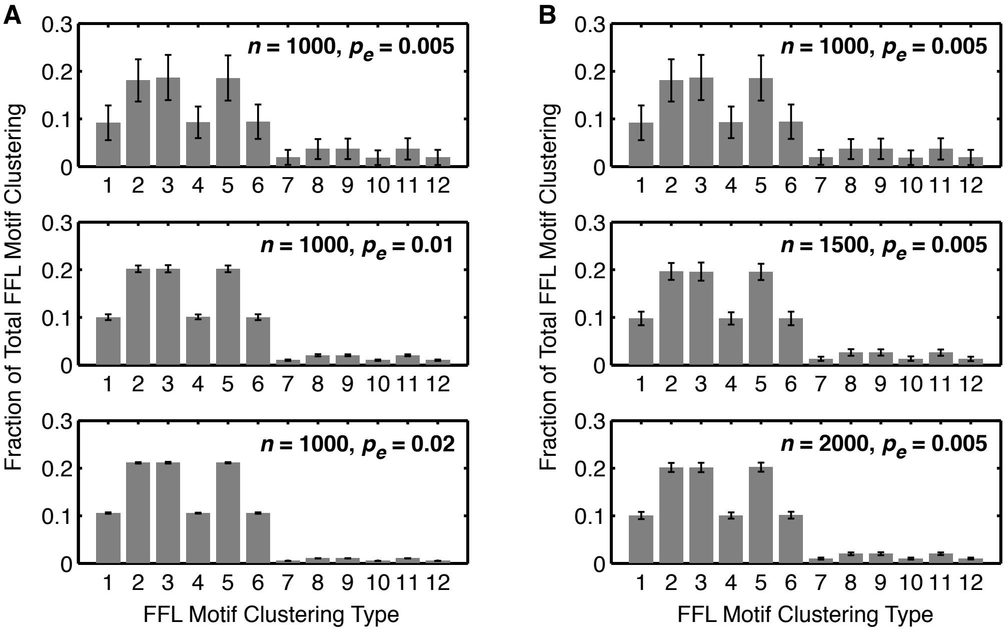

fig. S14. Feed-forward loop motif clustering type distributions for the Erdos-Renyí model. We analysed the effect of varying both network size n and edge probability p_e, and calculated 1000 random networks for each scenario. The specific FFL motif clustering types on the x-axis are shown in Fig. 3A in the main text. (**A**) Fixed size network of 1000 nodes and several edge probabilities of 0.005, 0.01 and 0.02. (**B**) Fixed edge probability of 0.005 and several network sizes of 1000, 1500 and 2000 nodes.

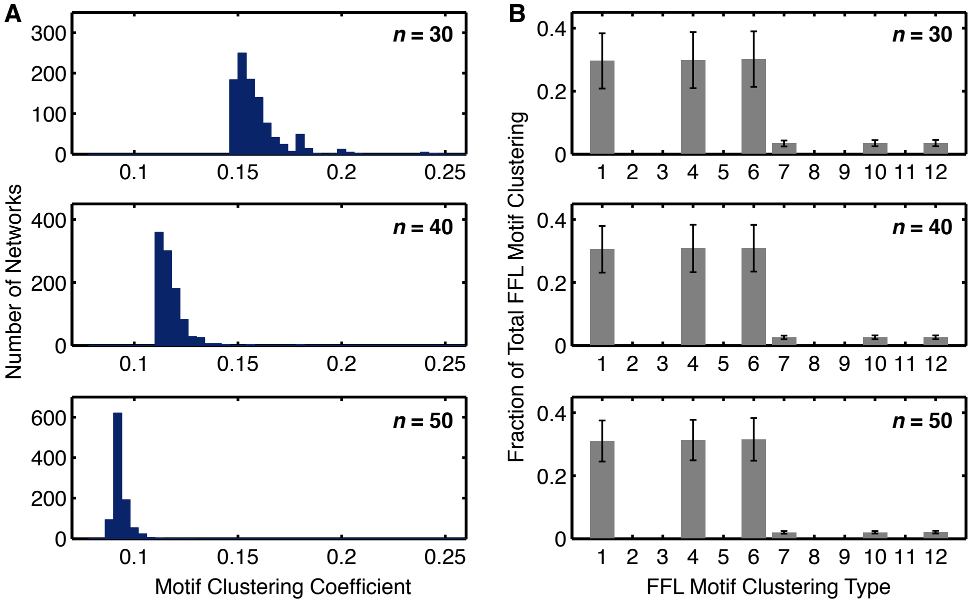

fig. S15. Motif clustering type distributions for the node duplication model. The size of the network was varied from 30 to 50 nodes and 1000 network samples were used. (**A**) Distribution of motif clustering coefficient for the samples. (**B**) Average motif clustering type distributions for the samples. Error bars denote $\pm$ one standard deviation.

table S1. General network statistics for the real-world systems. Diameter was calculated as the longest directed geodesic in the network and the standard clustering coefficient was generated by considering all edges as undirected.

**Network**	**Nodes**	**Edges**	**⟨Degree⟩**	**Density**	**Diameter**	**Clustering**
E. coli TF	424	519	1.22	0.0029	4	0.0238
S. cerevisiae TF	689	1079	1.56	0.0023	5	0.0162
E. coli Metabolism	561	9973	17.81	0.0319	12	0.4738
A.filgidus Metabolism	315	5434	17.25	0.0549	12	0.6121
C. elegans Neural	297	2345	7.90	0.0267	14	0.1807
Gnutella File Sharing	10879	39994	3.68	0.0003	26	0.0054
Air Traffic Control	1226	2613	2.13	0.0017	25	0.0639
Little Rock Lake	183	2476	13.53	0.0743	6	0.3323
EU E-mail	265214	364481	1.37	0.000005	16	0.0041
Wikipedia Vote	7115	103689	14.57	0.0020	10	0.1255

table S2. Motif related statistics for feed-forward loops (FFL) in the real-world networks. Random FFL counts and motif clustering values are presented with ± one standard deviation. Statistics were calculated from samples of 10000 randomised networks. EU E-mail and Wikipedia Vote networks have been omitted due to their size and density making sampling unfeasible.

	FFL Motif Counts (N)			FFL Motif Clustering (M_c)		
**Network**	**Real N**	**Random N**	**Z-score**	**Real** M_c	**Random** M_c	**Z-score**
E. coli TF	42	7.7 ± 3.2	10.9	0.136	0.0108 ± 0.0027	46.4
S. cerevisiae TF	70	13.8 ± 4.1	13.8	0.107	0.0065 ± 0.0011	88.5
E. coli Metabolism	78980	36125 ± 370	115.8	0.071	0.0094 ± 0.0001	485.7
A. fulgidus Metabolism	43282	22565 ± 234	88.3	0.116	0.0176 ± 0.0005	218.8
C. elegans Neural	3224	1694 ± 57	26.8	0.050	0.0187 ± 0.0004	71.41
Gnutella File Sharing	931	294.1 ± 17.4	36.6	0.004	0.0004 ± 0.00002	153.2
Air Traffic Control	326	31.9 ± 5.7	51.1	0.024	0.0037 ± 0.0004	47.0
Little Rock Lake	11289	10233 ± 115	9.1	0.106	0.0317 ± 0.0010	73.5

table S3. Statistics comparing the original and extracted FFLs for the real-world systems.

Network	Network Nodes			Network Edges		
	**Entire**	**FFL Extract**	**%**	**Entire**	**FFL Extract**	**%**
E. coli TF	424	67	15.8	519	102	19.7
S. cerevisiae TF	689	92	13.4	1079	153	14.2
E. coli Metabolism	561	411	73.3	9973	4182	41.9
A.filgidus Metabolism	315	187	59.4	5434	1872	34.4
C. elegans Neural	297	274	92.3	2359	1732	73.4
Gnutella File Sharing	10879	1676	15.4	39994	2335	5.8
Air Traffic Control	1226	233	19.0	2612	364	13.9
Little Rock Lake	183	99	54.1	2476	954	38.5
EU E-mail	265214	1224	0.5	64481	2955	0.8
Wikipedia Vote	7115	3897	54.8	103689	88682	85.5

table S4. Results for motif clustering in random network models. $\mathbb{M}_1$ and $\mathbb{M}_2$ denote the sum of pairs of motif that share 1 and 2 nodes respectively, see Fig. 3A in the main text for the specific motif clustering types. These results were calculated from 1000 computationally generated networks using two random network models. Averaged results from this sample are denoted by $\langle \cdot \rangle$ and we include $\pm$ the standard deviation. For comparison, column $\mathbb{P}$ shows the ratio $\mathbb{M}_1^{FFL}/\mathbb{M}_2^{FFL}$ predicted using the analytical results from this section.

**Model**	n	p_e	$\langle \mathbb{M}_1^{FFL} \rangle$	$\langle \mathbb{M}_2^{FFL} \rangle$	$\langle \mathbb{M}_1^{FFL}/\mathbb{M}_2^{FFL} \rangle$	$\mathbb{P}$
Erdős-Rényi	1000	0.005	68.2 ± 17.2	13.7 ± 4.6	5.38 ± 1.85	4.96
Erdős-Rényi	1000	0.01	$4.2\times10^3 \pm 393.7$	425.6 ± 42.0	9.88 ± 0.49	9.86
Erdős-Rényi	1000	0.02	$2.5\times10^5 \pm 1.3\times10^4$	$1.3\times10^4 \pm 577.7$	19.52 ± 0.22	19.52
Erdős-Rényi	1500	0.005	514.6 ± 64.5	69.2 ± 11.2	7.54 ± 0.96	7.44
Erdős-Rényi	2000	0.005	$2.2\times10^3 \pm 175.7$	219.5 ± 22.4	9.95 ± 0.73	9.93
Duplication	30	—	$1.1\times10^5 \pm 1.3\times10^4$	$1.2\times10^4 \pm 1.1\times10^3$	8.67 ± 0.32	8.67
Duplication	40	—	$4.9\times10^5 \pm 4.4\times10^4$	$4.1\times10^4 \pm 2.7\times10^3$	12.00 ± 0.31	12.0
Duplication	50	—	$1.6\times10^6 \pm 1.2\times10^5$	$1.0\times10^5 \pm 5.8\times10^3$	15.35 ± 0.32	15.3

table S5. Structural analysis of duplicated *E. coli* operon candidates. The structural superfamily classifications were carried out individually for each protein that the operon coded for (see UniProt IDs column) and the list of superfamily descriptions is given in order with each protein separated by a semicolon. Where possible we have included weak hits when no significant classification was found. Operons that share structural domain classifications in the same candidate set are denoted by †.

E. coli **Target Operon** Name	UniProt ID(s)	**Structural Domain Classifications** (Superfamily)
		**Candidate Set 1**
glpABC†	P0A9C0, P13033, P0A996	FAD/NAD(P)-binding domain; FAD/NAD(P)-binding domain; alpha-helical ferredoxin
icdA	P08200	Isocitrate/Isopropylmalate dehydrogenase-like
cydAB	P0ABJ9, P0ABK2	No significant hits; No significant hits
sdhCDAB† _b0725_suc ABCD	P69054, P0AC44, P0AC41, P07014, P0AFG3, P0AFG6, P0A836, P0AGE9	Fumarate reductase respiratory complex transmembrane subunits; Fumarate reductase respiratory complex transmembrane subunits; FAD/NAD(P)-binding domain AND Succinate dehydrogenase/fumarate reductase flavoprotein C-terminal domain AND Succinate dehydrogenase/fumarate reductase flavoprotein, catalytic domain; alpha-helical ferredoxin AND 2Fe-2S ferredoxin-like; Thiamin diphosphate-binding fold (THDP-binding) AND Thiamin diphosphate-binding fold (THDP-binding); CoA-dependent acyltransferases AND Single hybrid motif AND Peripheral subunit-binding domain of 2-oxo acid dehydrogenase complex; Glutathione synthetase ATP-binding domain-like AND Succinyl-CoA synthetase domains; Succinyl-CoA synthetase domains AND NAD(P)-binding Rossmann-fold domains
cyoABCDE	P0ABJ1, P0ABI8, P0ABJ3, P0ABJ6, P0AEA5	Cupredoxins AND Cytochrome c oxidase subunit II-like, transmembrane region; Cytochrome c oxidase subunit I-like; Cytochrome c oxidase subunit III-like; No significant hits; No significant hits (weak Multidrug efflux transporter AcrB transmembrane domain)
nuoABCEFG HIJKLMN	P0AFC3, P0AFC7, P33599, P0AFD1, P31979, P33602, P0AFD4, P0AFD6, P0AFE0, P0AFE4, P33607, P0AFE8, P0AFF0	No significant hits; HydA/Nqo6-like; HydB/Nqo4-like AND Nqo5-like; Thioredoxin-like; Nqo1 FMN-binding domain-like AND Nqo1C-terminal domain-like AND Nqo1 middle domain-like; Formate dehydrogenase/DMSO reductase, domains 1-3 AND 4Fe-4S ferredoxins AND 2Fe-2S ferredoxin-like AND ADC-like; No significant hits; 4Fe-4S ferredoxins; No significant hits; No significant hits; No significant hits; No significant hits; No significant hits
focA_pflB	P0AC23, P09373	No significant hits; PFL-like glycyl radical enzymes
		**Candidate Set 2**

fliFGHIJK	P25798, P0ABZ1, P31068, P52612, P52613, P52614	No significant hits; FliG AND FliG; No significant hits (weak V-type ATPase subunit E-like); P-loop containing nucleoside triphosphate hydrolases; No significant hits (weak Tropomyosin); No significant hits
fliMNOPQR	P06974, P15070, P22586, P0AC05, P0AC07, P33135	CheC-like AND Surface presentation of antigens (SPOA); Surface presentation of antigens (SPOA); No significant hits; No significant hits (weak Glycerol-3-phosphate (1)-acyltransferase); No significant hits (weak CytB endotoxin-like); No significant hits
flgBCDEFG HIJK	P0ABW9, P0ABX2, P75936, P75937, P75938, P0ABX5, P0A6S0, P0A6S3, P75942, P33235	No significant hits; No significant hits; No significant hits (weak Enolase N-terminal domain-like); Flagellar hook protein flgE; Flagellar hook protein flgE; Flagellar hook protein flgE; No significant hits; No significant hits; No significant hits (weak Lysozyme-like); Phase 1 flagellin
flhBAE	P76299, P76298, P76297	EscU C-terminal domain-like; No significant hits; No significant hits
fliE	P0A8T5	No significant hits (weak ATP synthase B chain-like)
	**Candidate Set 3**	
fumC	P05042	L-aspartase-like
nfo	P0A6C1	Xylose isomerase-like
zwf	P0AC53	Glyceraldehyde-3-phosphate dehydrogenase-like, C-terminal domain AND NAD(P)-binding Rossmann-fold domains
sodA	P00448	Fe,Mn superoxide dismutase (SOD), C-terminal domain and Fe AND Mn superoxide dismutase (SOD), N-terminal domain
	**Candidate Set 4**	
nac†	Q47005	Periplasmic binding protein-like II AND "Winged helix" DNA-binding domain
glnHPQ†	P0AEQ3, P0AEQ6, P10346	Periplasmic binding protein-like II; MetI-like; P-loop containing nucleoside triphosphate hydrolases
fdhF	P07658	Formate dehydrogenase/DMSO reductase, domains 1-3 AND ADC-like
hycABCDEF GH	P0AEV4, P0AAK1, P16429, P16430, P16431, P16432, P16433, P0AEV7	No significant hits; 4Fe-4S ferredoxins; No significant hits; No significant hits; HydB/Nqo4-like AND Nqo5-like; 4Fe-4S ferredoxins; HydA/Nqo6-like; No significant hits
	**Candidate Set 5**	
ompF†	P02931	Porins
ompC†	P06996	Porins
	**Candidate Set 6**	
malEFG	P0AEX9, P02916, P68183	Periplasmic binding protein-like II; MalF N-terminal region-like AND MetI-like; MetI-like
malS	P25718	(Trans)glycosidases

malK_lamB _malM	P68187, P02943, P03841	P-loop containing nucleoside triphosphate hydrolases AND MOP-like; Porins; No Significant Hits
**Candidate Set 7**		
manXYZ	P69797, P69801, P69805	PTS IIb component AND PTS system fructose IIA component-like; No significant hits; No significant hits
nagE	P09323	Duplicated hybrid motif AND Glucose permease domain IIB
**Candidate Set 8**		
fixABCX	P60566, P31574, P68644, P68646	Adenine nucleotide alpha hydrolases-like; DHS-like NAD/FAD-binding domain AND Adenine nucleotide alpha hydrolases-like; FAD/NAD(P)-binding domain AND FAD-linked reductases, C-terminal domain; 4Fe-4S ferredoxins
caiTABCDE	P31553, P60584, P31572, P31552, P31551, P39206	No significant hits; Acyl-CoA dehydrogenase NM domain-like AND Acyl-CoA dehydrogenase C-terminal domain-like; CoA-transferase family III (CaiB/BaiF); Acetyl-CoA synthetase-like; ClpP/crotonase; Trimeric LpxA-like enzymes
**Candidate Set 9**		
araBAD	P08204, P08202, P08203	2 x Actin-like ATPase domain; FucI/AraA N-terminal and middle domains AND FucI/AraA C-terminal domain-like; AraD/HMP-PK domain-like
araE†	P0AE24	MFS general substrate transporter
araJ†	P23910	MFS general substrate transporter
araFG_araH _1H_2	P02924, P0AAF3, P0AE26	Periplasmic binding protein-like I; P-loop containing nucleoside triphosphate hydrolases; No Significant Hits

table S6. Essential EC numbers for the *E. coli* metabolic network. The classification refer to the measures that predicted the node to be essential with 'D' degree, 'B' betweenness, and 'M' motif clustering diversity (MCD).

EC Number	Associated Gene	Degree	Betweenness	MCD	Classification
5.1.1.3	murI	52	0.00004	11	D
3.5.4.25	ribA	7	0.00427	6	B
2.7.7.2	ribF	43	0.00558	11	B
2.5.1.61	hemC	2	0.00508	0	B
2.7.8.5	pgsA	10	0.00299	8	B
2.7.7.41	cdsA	10	0.00377	5	B
2.6.1.16	glmS	37	0.00563	11	B
4.2.1.52	dapA	48	0.00596	11	B
2.1.2.1	glyA	31	0.00356	11	B
2.7.7.3	coaD	38	0.00212	12	M
6.3.2.13	murE	50	0.00105	12	M
2.3.1.51	plsC	29	0.00046	12	M
2.7.1.26	ribF	52	0.00506	11	DB
2.7.1.148	ispE	50	0.00343	8	DB
2.2.1.7	dxs	59	0.00824	11	DB
6.1.1.5	ileS	185	0.00275	12	DM
6.1.1.4	leuS	185	0.00275	12	DM
2.7.4.8	gmk	54	0.00246	12	DM
2.7.4.9	tmk	51	0.00205	12	DM
6.3.2.5	coaB	55	0.00104	12	DM
6.3.1.5	nadE	59	0.00142	12	DM
2.7.6.3	folK	60	0.00175	12	DM
6.3.4.15	birA	62	0.00197	12	DM
2.4.1.227	murG	24	0.00501	12	BM
6.1.1.7	alaS	190	0.01135	12	DBM
6.1.1.19	argS	186	0.00417	12	DBM
6.1.1.12	aspS	192	0.00909	12	DBM
6.1.1.16	cysS	192	0.00669	12	DBM
6.1.1.17	gltX	228	0.02937	12	DBM
6.1.1.21	hisS	187	0.01161	12	DBM
6.1.1.10	metG	188	0.00753	12	DBM
6.1.1.15	proS	187	0.00721	12	DBM
6.1.1.11	serS	192	0.01389	12	DBM
6.1.1.3	thrS	188	0.00768	12	DBM
6.1.1.2	trpS	185	0.00774	12	DBM
6.1.1.1	tyrS	185	0.00300	12	DBM
6.1.1.9	valS	186	0.00353	12	DBM
2.7.7.8	pnp	105	0.00543	12	DBM
6.3.4.2	pyrG	87	0.00386	12	DBM

2.7.4.6	ndk	256	0.06379	12	DBM
2.7.1.24	coaE	70	0.00565	12	DBM
2.5.1.6	metK	159	0.02168	12	DBM
6.3.4.14	accC	230	0.01551	12	DBM
6.3.2.8	murC	54	0.00406	12	DBM
2.7.2.3	pgk	234	0.02696	12	DBM
2.7.2.1	ackA	246	0.03770	12	DBM
2.7.6.1	prs	64	0.00462	12	DBM
2.1.2.9	fmt	18	0.00024	7	None
2.1.1.45	thyA	15	0.00114	6	None
1.1.1.193	ribD	2	0.00171	0	None
3.5.4.26	ribD	1	0.00000	0	None
4.1.1.36	coaB	3	0.00000	10	None
2.3.1.39	fabD	28	0.00068	11	None
2.5.1.10	ispA	4	0.00000	0	None
2.5.1.31	uppS	3	0.00000	0	None
1.1.1.49	zwf	4	0.00000	6	None
1.8.1.4	lpdA	9	0.00022	11	None
2.7.7.23	glmU	8	0.00053	8	None
1.2.1.11	asd	2	0.00000	0	None
1.3.1.26	dapB	3	0.00003	0	None
1.5.1.2	proC	6	0.00000	0	None
